# Supplementary material for: Simulating Chern insulators on a superconducting quantum processor
Source: Nat Commun. 2023 Sep 5;14:5433. doi: 10.1038/s41467-023-41230-9 (PMC10480218; doi:10.1038/s41467-023-41230-9)
Supplement: Supplementary file 1 — Supplementary Information [file 41467_2023_41230_MOESM1_ESM.pdf]

Supplementary Information for:
Simulating Chern insulators on a superconducting quantum processor

Zhong-Cheng Xiang,[1,*] Kaixuan Huang,[2,1,3,*] Yu-Ran Zhang,[4,5,6,*] Tao Liu,[4] Yun-Hao Shi,[1] Cheng-Lin Deng,[1] Tong Liu,[1] Hao Li,[1] Gui-Han Liang,[1] Zheng-Yang Mei,[1] Haifeng Yu,[2] Guangming Xue,[2] Ye Tian,[1] Xiaohui Song,[1] Zhi-Bo Liu,[3] Kai Xu,[1,2,7,8,†] Dongning Zheng,[1,7,8] Franco Nori,[5,6,9,‡] and Heng Fan[1,2,7,8,§]

[1]*Institute of Physics, Chinese Academy of Sciences, Beijing 100190, China*
[2]*Beijing Academy of Quantum Information Sciences, Beijing 100193, China*
[3]*Key Laboratory of Weak Light Nonlinear Photonics, Ministry of Education, Teda Applied Physics Institute and School of Physics, Nankai University, Tianjin 300457, China*
[4]*School of Physics and Optoelectronics, South China University of Technology, Guangzhou 510640, China*
[5]*Theoretical Quantum Physics Laboratory, Cluster for Pioneering Research, RIKEN, Wako-shi, Saitama 351-0198, Japan*
[6]*Center for Quantum Computing, RIKEN, Wako-shi, Saitama 351-0198, Japan*
[7]*CAS Centre for Excellence in Topological Quantum Computation, UCAS, Beijing 100190, China*
[8]*Songshan Lake Materials Laboratory, Dongguan 523808, China*
[9]*Physics Department, University of Michigan, Ann Arbor, Michigan 48109-1040, USA*

CONTENTS

Supplementary Note 1. Experimental setup 1
- A. Fabrication 1
- B. Wiring 1
- C. Device information 2
- D. System Hamiltonian 3
- E. Qubits coupling strengths 3
- F. Single-qubit gates 4

Supplementary Note 2. Calibration 6
- A. Pulse amplitudes for Z and XY controls 6
- B. Readout probability 6
- C. Z pulse distortion 6
- D. Z pulse crosstalk 7
- E. Qubits frequencies 8
- F. Phase calibration 9

Supplementary Note 3. Supplementary experimental data 10
- A. Single-excitation quantum walks 10
- B. Band structure spectroscopy 11
- C. Quantum charge pumping and fast pumping 13
- D. Numerical details 13

References 13

* These authors contributed equally to this work.
† kaixu@iphy.ac.cn
‡ fnori@riken.jp
§ hfan@iphy.ac.cn

Supplementary Note 1. EXPERIMENTAL SETUP

A. Fabrication

Our experiments are performed on a superconducting circuit consisting of 30 transmon qubits ($Q_{j,s}$, with j varied from 1 to 15 and pseudo-spin $s = \{\uparrow, \downarrow\}$), which constitute a two-legged qubit ladder, as shown in Supplementary Fig. 1. Each qubit, coupled to an independent readout resonator (R), has an independent microwave line for XY and Z controls. This ladder-type 30-superconducting-qubit device is fabricated on a 430 μm thick sapphire chip with standard wafer cleaning.

First, a 100 nm thick layer of Al is deposed on a 10 $\times$ 10 mm^2 sapphire substrate, which is patterned with optical lithography using a 0.70 μm thick layer of positive SPR955 resist. Then, we apply the wet etching method to produce the large components of the superconducting chip, such as the microwave coplanar waveguide resonators, the transmission lines, the control lines, and the capacitors of the transmon qubits.

The second step is the preparation process for Josephson junctions. After patterning a bilayer of MMA and PMMA resists with electron beam lithography, the Josephson junctions are made using double-angle evaporations, which include a 65 nm thick Al layer at $+60^\circ$ followed by an oxidation process in pure oxygen for several minutes and then a 100 nm thick second layer of Al at 0°. Finally, in order to suppress the parasitic modes, several airbridges [1] are constructed on the chip.

B. Wiring

A schematic diagram of our experimental setup and wiring is shown in Supplementary Fig. 2. In our experiments, the superconducting quantum processor is cooled in a dilution refrigerator (BlueFors, XLD1000sl) with a base temperature of mixing chamber (MC) about 15 mK. This device has three readout lines, and the readout signals are amplified by the Josephson-based parametric amplifiers (JPAs) and the high-

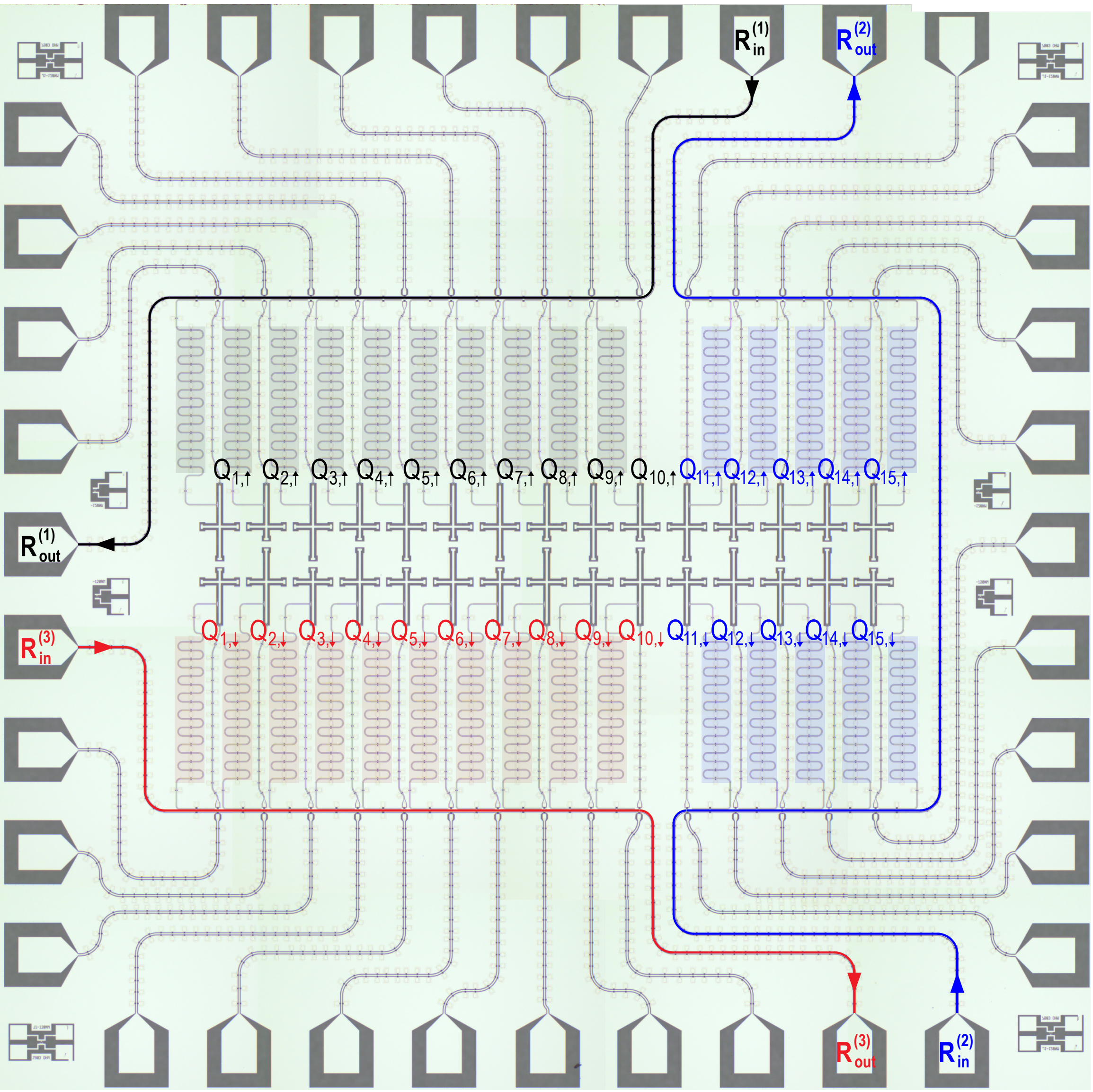

Supplementary Fig. 1. Optical micrograph of the thirty-qubit sample. Thirty superconducting qubits constitute a ladder. Each qubit has an independent readout resonator and an independent microwave line for XY and Z controls. Thirty readout resonators are separated into three groups, and ten resonators in each group share a microwave transmission line (highlighted in black, blue or red) for measurements.

electron-mobility transistors (HEMTs). The specific arrangement of the attenuators and filters are used for noise suppression. The up-conversion principle is used to generate the XY and read-in signals, while the down-conversion principle is for readout signal. The Z signals are directly generated by the arbitrary waveform generator (AWG). The XY and Z signals are coupled together at the MC plate of the dilution refrigerator by using a directional coupler.

C. Device information

For the 15-qubit experiment, fifteen superconducting qubits ($Q_{j,\uparrow}$ with j from 1 to 15) on one leg of the qubit-ladder are used, which constitutes a one-dimensional (1D) qubit-chain. The idle frequencies of 15 qubits, denoted by $\omega^{\mathrm{idle}}_{j,\uparrow}/2\pi$, are carefully arranged to minimise the crosstalk error among qubits during the single-qubit operations, while the 15 unused qubits ($Q_{j,\downarrow}$ with j from 1 to 15) on the other leg are detuned far from 3.5 GHz to avoid any unwanted interaction. The qubits characteristics for the 15-qubit experiment are shown in Supplementary Table 1.

For the 30-qubit experiment, all thirty superconducting qubits ($Q_{j,s}$ with j from 1 to 15 and $s \in \{\uparrow, \downarrow\}$) are used. The qubit's characteristics for the 30-qubit experiment are shown in Supplementary Table 2. As shown in Supplementary Table 2, the decoherence times T^1 and T^{2*} are much longer than the system's evolution time within 1 μs.

	$\omega^{\rm idle}_{j,\uparrow}/2\pi$ (GHz)	$\omega^{\rm max}_{j,\uparrow}/2\pi$ (GHz)	$\bar{T}^1_{j,\uparrow}$ (μs)	$\omega^{\rm R}_j/2\pi$ (GHz)	$g_j/2\pi$ (MHz)	$\eta_j/2\pi$ (GHz)	$F^0_{j,\uparrow}$	$F^1_{j,\uparrow}$
$Q_{1,\uparrow}$	4.410	4.879	∼30	6.623	35.82	0.203	0.975	0.937
$Q_{2,\uparrow}$	5.290	5.384	∼27	6.640	37.57	0.252	0.977	0.916
$Q_{3,\uparrow}$	4.517	4.921	∼32	6.659	33.85	0.202	0.975	0.920
$Q_{4,\uparrow}$	5.370	5.387	∼30	6.677	36.25	0.246	0.978	0.921
$Q_{5,\uparrow}$	4.360	4.910	∼41	6.703	35.41	0.202	0.977	0.927
$Q_{6,\uparrow}$	5.230	5.458	∼30	6.720	41.83	0.250	0.974	0.932
$Q_{7,\uparrow}$	4.499	4.821	∼40	6.739	36.30	0.204	0.961	0.897
$Q_{8,\uparrow}$	5.320	5.541	∼33	6.758	34.07	0.247	0.963	0.916
$Q_{9,\uparrow}$	4.430	5.027	∼39	6.777	33.82	0.200	0.955	0.912
$Q_{10,\uparrow}$	5.150	5.447	∼37	6.803	38.06	0.246	0.983	0.885
$Q_{11,\uparrow}$	4.480	4.989	∼38	6.618	35.80	0.200	0.967	0.901
$Q_{12,\uparrow}$	5.350	5.420	∼30	6.635	39.11	0.251	0.969	0.910
$Q_{13,\uparrow}$	4.335	5.046	∼39	6.656	31.94	0.202	0.971	0.883
$Q_{14,\uparrow}$	5.260	5.462	∼30	6.671	35.10	0.244	0.977	0.895
$Q_{15,\uparrow}$	4.450	5.070	∼31	6.695	33.90	0.202	0.979	0.886

Supplementary Table 1. Qubit characteristics for the 15-qubit experiment using 15 qubits (from $Q_{1,\uparrow}$ to $Q_{15,\uparrow}$) on the same leg. $\omega^{\rm idle}_{j,\uparrow}/2\pi$ is the idle frequency of the qubit, at which the single-qubit gates are performed. $\bar{T}^1_{j,\uparrow}$ denotes the average energy relaxation time from the frequency about 4.0 GHz to the maximum frequency $\omega^{\rm max}_{j,\uparrow}/2\pi$ of Q_j. Also, $\omega^{\rm R}_{j,\uparrow}/2\pi$ is the readout resonator's frequency of $Q_{j,\uparrow}$ with $g_{j,\uparrow}/2\pi$ being the coupling strength between the qubit and the readout resonator. The anharmonicity $\eta_{j,\uparrow}/2\pi$ of $Q_{j,\uparrow}$ is defined by $\eta_{j,\uparrow} \equiv \omega^{10}_{j,\uparrow} - \omega^{21}_{j,\uparrow}$, with $\omega^{10}_{j,\uparrow}/2\pi$ ($\omega^{21}_{j,\uparrow}/2\pi$) being the energy difference between the $|1\rangle$ and $|0\rangle$ states ($|2\rangle$ and $|1\rangle$ states). $F^0_{j,\uparrow}$ ($F^1_{j,\uparrow}$) is the measurement probability of $|0\rangle$ ($|1\rangle$) when the qubit is prepared at $|0\rangle$ ($|1\rangle$), which is used to mitigate the readout errors.

D. System Hamiltonian

Our superconducting quantum processor can be described as a Bose-Hubbard ladder with a Hamiltonian ($\hbar = 1$) [2, 3]

$$H_{\rm BH} = J_{\parallel}\sum_{j,s}(\hat{a}^{\dagger}_{j,s}\hat{a}_{j+1,s} + \text{H.c.}) + \sum_{j,s}\frac{\eta_{j,s}}{2}\hat{n}_{j,s}(\hat{n}_{j,s}-1) + J_{\perp}\sum_{j}(\hat{a}^{\dagger}_{j,\uparrow}\hat{a}_{j,\downarrow} + \text{H.c.}) + \sum_{j,s}V_{j,s}\hat{n}_{j,s}, \quad (1)$$

where $\hat{a}^{\dagger}$ ($\hat{a}$) is the bosonic creation (annihilation) operator, and $\hat{n} \equiv \hat{a}^{\dagger}\hat{a}$ is the number operator. Here, $J_{\parallel}/2\pi \simeq 8$ MHz and $J_{\perp}/2\pi \simeq 7$ MHz denote the nearest-neighbour (NN) hopping between nearby qubits on the same leg and on the same rung, respectively. Also, η is the on-site nonlinear interaction, and $V_{j,s}$ is the tuneable on-site potential.

Our device is designed to fulfil the hard-core limit $|\eta/J| \gg 1$, and thus, the highly occupied states of transmon qubits are blockaded, which represents the fermionisation of strongly interacting bosons [4]. The system Hamiltonian can then be simplified as

$$H = J_{\parallel}\sum_{j,s}(\hat{c}^{\dagger}_{j,s}\hat{c}_{j+1,s} + \text{H.c.}) + \sum_{j,s}V_{j,s}\hat{c}^{\dagger}_{j,s}\hat{c}_{j,s} + J_{\perp}\sum_{j}(\hat{c}^{\dagger}_{j,\uparrow}\hat{c}_{j,\downarrow} + \text{H.c.}), \quad (2)$$

where $\hat{c}^{\dagger}$ ($\hat{c}$) is the hard-core bosonic creation (annihilation) operator with $(\hat{c}^{\dagger})^2 = \hat{c}^2 = 0$, and $[\hat{c}^{\dagger}_{j,s}, \hat{c}_{i,r}] = \delta_{ji}\delta_{sr}$.

Note that in addition to the hopping between nearest-neighbour (NN) qubits, there also exist the hopping between next-nearest-neighbour (NNN) qubits on different legs:

$$J_{\times}\sum_{j}(\hat{c}^{\dagger}_{j,\uparrow}\hat{c}_{j+1,\downarrow} + \hat{c}^{\dagger}_{j,\downarrow}\hat{c}_{j+1,\uparrow} + \text{H.c.}), \quad (3)$$

and the hopping between third-nearest-neighbour (TNN) qubits on the same leg:

$$J'_{\parallel}\sum_{j,s}(\hat{c}^{\dagger}_{j,s}\hat{c}_{j+2,s} + \text{H.c.}). \quad (4)$$

E. Qubits coupling strengths

The coupling strengths between the nearest-neighbour (NN), next-nearest-neighbour (NNN), and third-nearest-neighbour (TNN) qubits are shown in Supplementary Fig. 3a and 3b for the 15-qubit and 30-qubit experiments, respectively. The coupling strengths between the selected qubits are measured at 4.7 and 4.5 GHz for the 15-qubit and 30-qubit experiments, respectively. For the 15-qubit experiment, we only measured the nearest and next-nearest coupling strengths, which are considered in the numerical simulation, since the TNN coupling in the 15-qubit chain is too weak and can be neglected. For the 30-qubit experiment, we measured the NN, NNN and TNN coupling strengths, which are used in the numerical simulation.

	$\omega^{\rm idle}_{j,s}/2\pi$ (GHz)	$\omega^{\rm max}_{j,s}/2\pi$ (GHz)	$\bar{T}^{1}_{j,s}$ (μs)	$T^{2*}_{j,s}$ (μs)	$\omega^{\rm R}_{j,s}/2\pi$ (GHz)	$g_{j,s}/2\pi$ (MHz)	$\eta_{j,s}/2\pi$ (GHz)	$F^{0}_{j,s}$	$F^{1}_{j,s}$
$Q_{1,\uparrow}$	4.050	4.879	∼30	∼1.2	6.623	35.82	0.203	0.891	0.902
$Q_{2,\uparrow}$	5.350	5.384	∼27	∼3.7	6.640	37.57	0.252	0.973	0.886
$Q_{3,\uparrow}$	4.090	4.921	∼32	∼0.9	6.659	33.85	0.202	0.924	0.883
$Q_{4,\uparrow}$	5.385	5.387	∼30	∼4.0	6.677	36.25	0.246	0.965	0.891
$Q_{5,\uparrow}$	4.460	4.910	∼41	∼4.6	6.703	35.41	0.202	0.947	0.870
$Q_{6,\uparrow}$	5.450	5.458	∼30	∼4.6	6.720	41.83	0.250	0.967	0.894
$Q_{7,\uparrow}$	4.065	4.821	∼40	∼3.1	6.739	36.30	0.204	0.911	0.877
$Q_{8,\uparrow}$	5.410	5.541	∼33	∼3.3	6.758	34.07	0.247	0.973	0.884
$Q_{9,\uparrow}$	4.175	5.027	∼39	∼2.7	6.777	33.82	0.200	0.931	0.842
$Q_{10,\uparrow}$	5.140	5.447	∼37	∼4.4	6.803	38.06	0.246	0.966	0.900
$Q_{11,\uparrow}$	4.405	4.989	∼38	∼1.3	6.618	35.80	0.200	0.908	0.853
$Q_{12,\uparrow}$	5.310	5.420	∼30	∼3.3	6.635	39.11	0.251	0.981	0.893
$Q_{13,\uparrow}$	4.100	5.046	∼39	∼2.4	6.656	31.94	0.202	0.925	0.880
$Q_{14,\uparrow}$	5.180	5.462	∼30	∼2.9	6.671	35.10	0.244	0.974	0.894
$Q_{15,\uparrow}$	4.530	5.070	∼31	∼4.1	6.695	33.90	0.202	0.946	0.880
$Q_{15,\downarrow}$	5.370	5.413	∼28	∼6.2	6.725	35.80	0.246	0.984	0.905
$Q_{14,\downarrow}$	4.390	4.926	∼38	∼1.6	6.746	36.64	0.198	0.949	0.902
$Q_{13,\downarrow}$	5.240	5.326	∼26	∼4.0	6.769	40.11	0.246	0.978	0.859
$Q_{12,\downarrow}$	4.350	4.905	∼40	∼1.9	6.791	35.97	0.198	0.950	0.896
$Q_{11,\downarrow}$	5.438	5.486	∼29	∼3.0	6.812	38.98	0.244	0.972	0.914
$Q_{10,\downarrow}$	4.485	5.087	∼38	∼1.2	6.628	32.70	0.202	0.963	0.875
$Q_{9,\downarrow}$	4.985	5.421	∼36	∼1.6	6.640	32.02	0.245	0.977	0.902
$Q_{8,\downarrow}$	4.445	4.930	∼40	∼1.9	6.665	35.24	0.203	0.958	0.872
$Q_{7,\downarrow}$	5.305	5.371	∼39	∼1.9	6.684	35.47	0.250	0.947	0.897
$Q_{6,\downarrow}$	4.503	4.968	∼28	∼1.7	6.710	36.88	0.200	0.925	0.904
$Q_{5,\downarrow}$	5.075	5.518	∼34	∼1.6	6.731	41.09	0.242	0.952	0.871
$Q_{4,\downarrow}$	4.365	4.976	∼41	∼1.2	6.751	37.09	0.201	0.951	0.864
$Q_{3,\downarrow}$	4.870	5.289	∼35	∼1.7	6.769	38.91	0.248	0.982	0.904
$Q_{2,\downarrow}$	4.323	4.922	∼31	∼1.5	6.791	34.72	0.203	0.939	0.891
$Q_{1,\downarrow}$	4.925	5.390	∼28	∼1.5	6.811	42.20	0.251	0.962	0.927

Supplementary Table 2. Qubit characteristics for the 30-qubit experiment using all 30 qubits ($Q_{j,s}$ with j from 1 to 15 and $s \in \{\uparrow,\downarrow\}$). $\omega^{\rm idle}_{j,s}/2\pi$ is the idle frequency of the qubit, where single-qubit gates are performed. $\bar{T}^{1}_{j,s}$ denotes the average energy relaxation time from the frequency about 4.0 GHz to the maximum frequency $\omega^{\rm max}_{j,s}/2\pi$ of Q_j. The dephasing time T_2^* is measured at the idle frequency $\omega^{\rm idle}_{j,s}/2\pi$. Also, $\omega^{\rm R}_{j,s}/2\pi$ is the readout resonator's frequency of $Q_{j,s}$ with $g_{j,s}/2\pi$ being the coupling strength between the qubit and the readout resonator. The anharmonicity $\eta_{j,s}/2\pi$ of $Q_{j,s}$ is defined by $\eta_{j,s} \equiv \omega^{10}_{j,s} - \omega^{21}_{j,s}$, with $\omega^{10}_{j,s}/2\pi$ ($\omega^{21}_{j,s}/2\pi$) being the energy difference between the $|1\rangle$ and $|0\rangle$ states ($|2\rangle$ and $|1\rangle$ states). $F^{0}_{j,s}$ ($F^{1}_{j,s}$) is the measurement probability of $|0\rangle$ ($|1\rangle$) when the qubit is prepared at $|0\rangle$ ($|1\rangle$), which is used to mitigate the readout errors.

F. Single-qubit gates

In our experiments, we initialise a qubit from the $|0\rangle$ state to the $|1\rangle$ and $(|0\rangle + |1\rangle)/\sqrt{2}$ states by applying a X_π and $Y_{\frac{\pi}{2}}$ gates on this qubit for quantum walks and topological band structure measurements, respectively. Single-qubit operations, including the $Y_{\frac{\pi}{2}}$ and X_π gates, have a duration of 120 ns and a full width at half maximum (FWHM) of 60 ns. By using the derivative removal by adiabatic gate (DRAG) theory, the quadrature correction terms with a DRAG coefficient are optimised to minimise the leakage to higher energy levels [5]. The readout pulses for all qubits have a duration of 2.0 μs, and the readout pulses powers and frequencies are optimised to realise high-visibility and low-error readouts. The readout fidelities, denoted by F_0 and F_1, are shown in Supplementary Table 1 and Supplementary Table 2 for the 15-qubit and 30-qubit experiments, respectively.

Here, to identify the accuracy of the initial state preparation, we calculate the fidelity of the prepared states compared with the perfect $|1\rangle$ and $(|0\rangle + |1\rangle)/\sqrt{2}$ states, respectively, by using the quantum state tomography (QST) technique. The QST measurements of the prepared states require individually measuring the qubit in bases formed by the eigenvectors of $\hat{\sigma}^x$, $\hat{\sigma}^y$, and $\hat{\sigma}^z$, respectively. In our experiments, the mea-

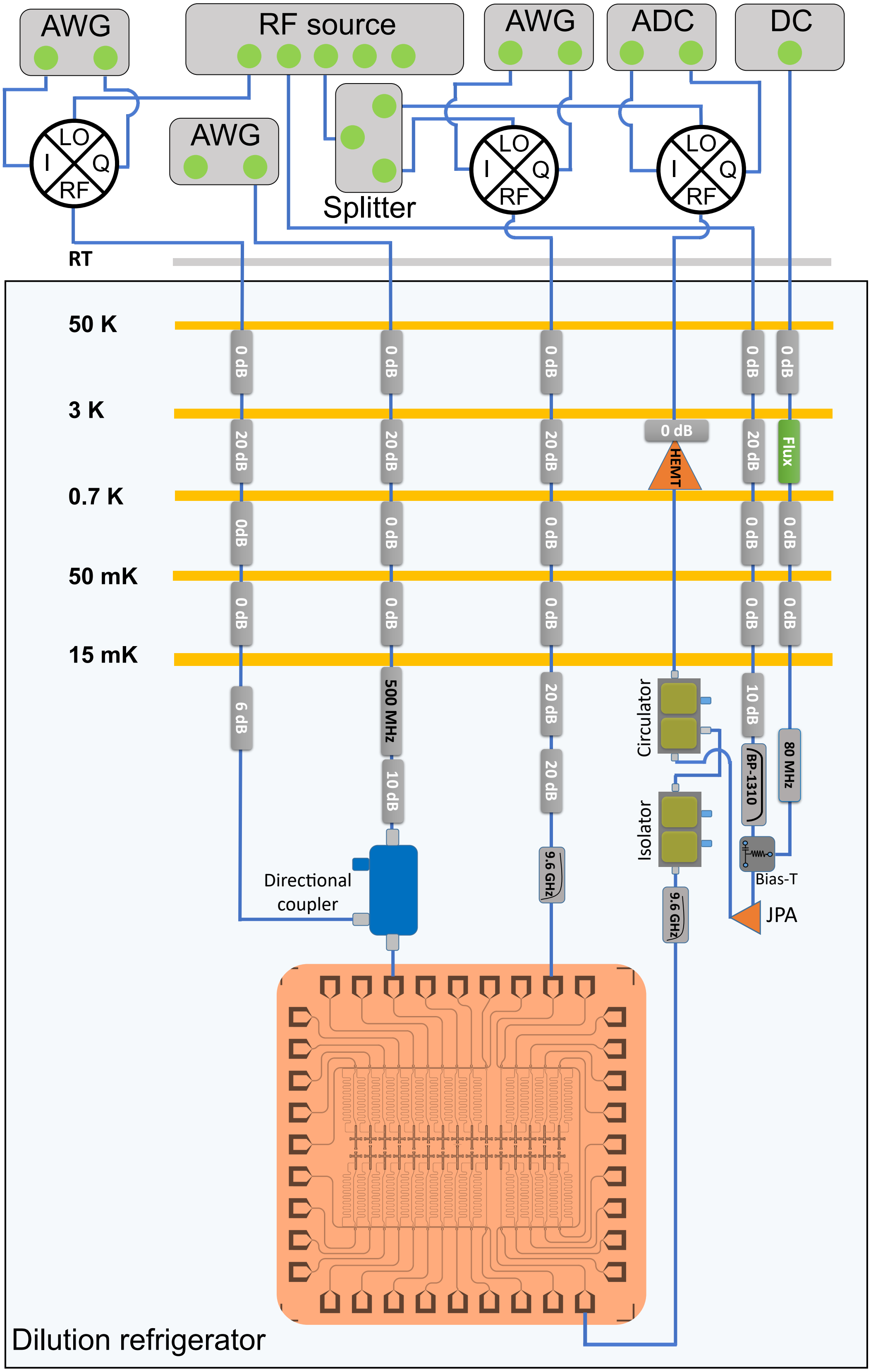

Supplementary Fig. 2. Experimental setup and low-temperature wiring.

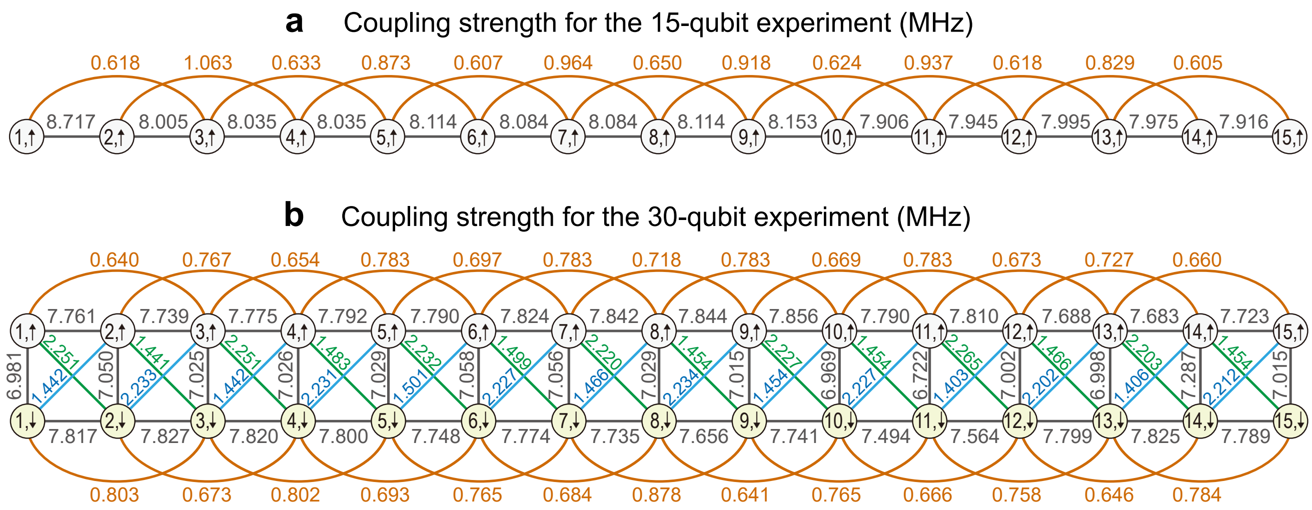

Supplementary Fig. 3. Experimental measured coupling strengths between nearest-neighbour (NN), next-nearest-neighbour (NNN), and third-nearest-neighbour (TNN) qubits, which are used for the numerical simulations of the 15-qubit experiment (a), and the 30-qubit experiment (b).

surement along the z-axis of the Bloch sphere can be directly performed, and those along the x- and y-axes are realised by inserting the $\mathrm{Y}_{-\frac{\pi}{2}}$ and $\mathrm{X}_{-\frac{\pi}{2}}$ gates on the qubit before the measurements, respectively. In total, we have three tomographic operations $\{\mathrm{I}, \mathrm{X}_{-\frac{\pi}{2}}, \mathrm{Y}_{-\frac{\pi}{2}}\}$ and obtain two occupation probabilities $\{P_0, P_1\}$ for each operation, which allow us to reconstruct the density matrix ρ_{exp} of the initially prepared state.

The fidelity between the experimentally measured density matrix ρ_{exp} and the ideal one $|\psi\rangle$ can be quantified as $F(\rho_{\text{exp}}, |\psi\rangle) = \langle\psi|\rho_{\text{exp}}|\psi\rangle$. We maintain a fixed sample of 3,000 repetitions of readouts and repeat the measurement procedure ten times for estimating the average value and the standard deviation of the state fidelity for each qubit. Experimental results of the fidelity for the $(|0\rangle + |1\rangle)/\sqrt{2}$ and $|1\rangle$ states are both above 0.990, which are shown in Supplementary Fig. 4a and 4b, respectively.

Supplementary Note 2. CALIBRATION

A. Pulse amplitudes for Z and XY controls

In our experiments, we periodically calibrate the amplitude of the Z pulse (about 10 μs) at the idle frequency and the pulse amplitude of the single-qubit gate on each qubit to avoid the variations of the performances of the Z and XY controls with time.

B. Readout probability

We monitor the time evolution of the readout fidelity during the experiment in order to perform the real-time correction of the readout errors [6], by applying the inverse of the tensor product of the matrices

$$\begin{pmatrix} F^0_{j,s} & 1 - F^1_{j,s} \\ 1 - F^0_{j,s} & F^1_{j,s} \end{pmatrix} \tag{5}$$

for the selected qubit $\mathrm{Q}_{j,s}$ to the measured occupation probability vector $(P^0_{j,s}, P^1_{j,s})^T$.

C. Z pulse distortion

In the waveform of the Z pulse to adjust the frequency of a qubit, the rising edge, the falling edge, and the flatness of the Z pulse are very significant for realising a high-fidelity quantum gate and the long-time quantum state evolution. The Z pulse distortion would cause an unwanted frequency drift before the readout process. This issue causes errors when we measure in the $\hat{\sigma}^x$ and $\hat{\sigma}^y$ bases, because the frequency drift would generate an unwanted phase shift that greatly affects the measured result [7]. Therefore, it is of fundamental importance to correct the unwanted Z pulse distortion. In our experiments, we develop two types of pulse sequences, which aim to correct the Z pulse distortions occurring in the short-time and long-time scales, respectively.

The existence of the DC component in the waveform of the Z pulse will shorten the dephasing time T^{2*} of the qubit. In our experiments, a DC-block element (with a capacitance $\sim$ 1–2μF) is employed for blocking the DC component. However, the use of the DC-block element inevitably distorts the shape of the Z pulse with a long time (over 500 ns), which decreases the modulation precision of the qubits frequencies.

Thus, we design a pulse sequence for correcting the Z pulse distortion as shown in Supplementary Fig. 5a. First, a X_π pulse around the target frequency and a Z pulse with a fixed duration and a fixed amplitude are applied to the qubit. Then, we vary the starting time of the X_π pulse and measure the

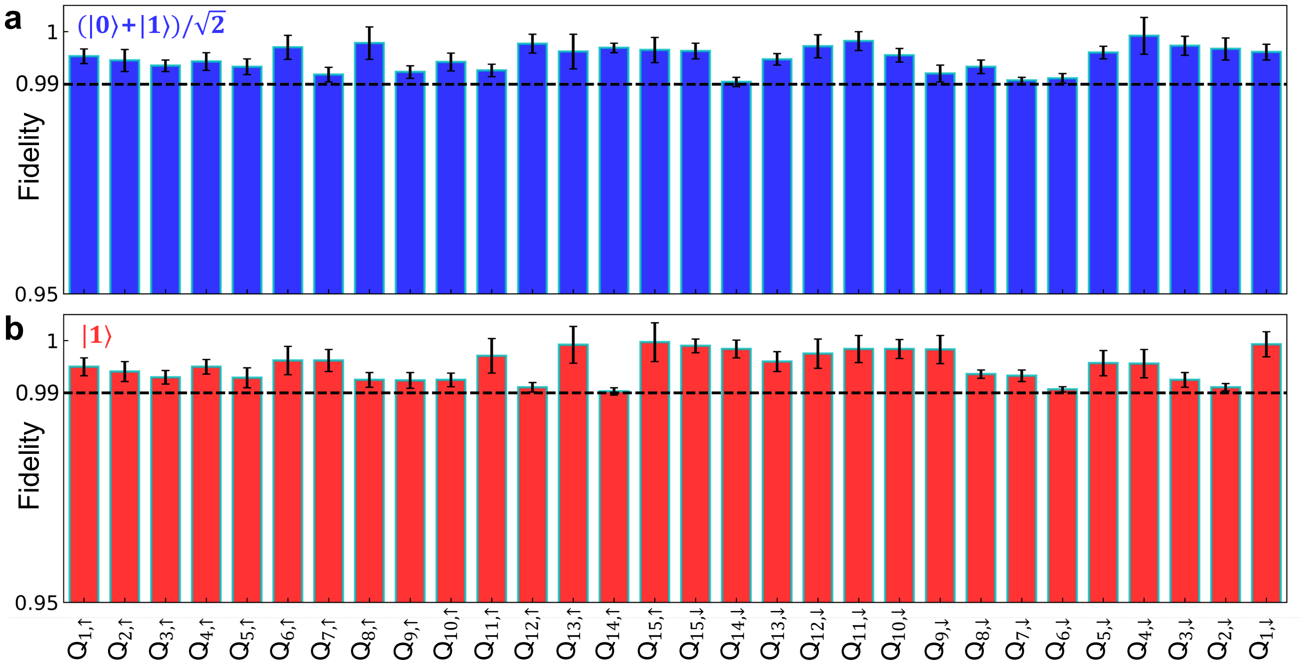

Supplementary Fig. 4. Single-qubit quantum state tomography (QST) for the $(|0\rangle + |1\rangle)/\sqrt{2}$ state (a) and the $|1\rangle$ state (b), which are prepared by applying $\mathrm{Y}_{\frac{\pi}{2}}$ and X_{π} gates, respectively.

population of the $|1\rangle$ state for different time delays. The qubit can be excited to $|1\rangle$ when the frequency of the qubit matches that of the applied X_π pulse. As shown in Supplementary Fig. 5c, the measured population of $|1\rangle$ before the Z correction shows the shape of the Z pulse that is sensed by the qubit. After obtaining the maximum value of the population of $|1\rangle$ for each delay, we can fit the data of the Z pulse amplitude (Zpa) with an exponential decay function:

$$P_1(t) = \alpha[1 + \exp(-t/T_\mathrm{d})] + \beta, \tag{6}$$

with α and β being the fitting parameters and T_0 being the estimated input of the characteristic decay factor T_d. As shown in Supplementary Fig. 5b, $T_\mathrm{d} = 155.45$ μs is fitted for the DC-block element with a capacitance 2 μF. With the obtained characteristic decay factor and the deconvolution method, we can send a corrected waveform to the arbitrary waveform generator (AWG). Finally, the qubit can sense a relatively flat Z pulse as shown in Supplementary Fig. 5d.

The above procedure can correct the distorted Z pulse for the long-time scale. To calibrate the Z pulse distortion within a short-time scale (e.g., $<$ 100 ns), the pulse sequence as shown in Supplementary Fig. 6a is designed to record the shape of the tail of the Z pulse. Note that the measurement is performed at the idle frequency of the qubit. Therefore, it is better to bias the qubit to a low frequency (1 GHz below the sweet point $\omega_{j,s}^{\max}/2\pi$) for obtaining a Zpa-sensitive region. The experimental result of the pulse-shape measurement is shown in Supplementary Fig. 6b, and an obvious distortion is observed before the correction. In our experiments, a finite impulse response (FIR) filter and several first-order infinite impulse response (IIR) filters are employed for the correction [8]. The FIR filter can be described by a polynomial function with 16–24 parameters, while the IIR filters are an integration of several exponential functions. The experimental result after the first round of the correction is shown in Supplementary Fig. 6c. Generally, in order to obtain a better performance, we repeat the correction procedure twice and finally obtain a stationary step response behaviour as shown in Supplementary Fig. 6d.

D. Z pulse crosstalk

The Z pulse crosstalks between different qubit pairs is calibrated by experimentally measuring the Z-crosstalk matrix $\tilde{M}_Z$ using a similar method as shown in Supplementary Ref. [9]. Each element of $\tilde{M}_Z$ denotes the Z pulse amplitude (Zpa) that is sensed by $\mathrm{Q}_{j,s}$, when a unity Zpa (1 arb. units) is applied to $\mathrm{Q}_{i,r}$. Note that all elements $(\tilde{M}_Z)_{i,r;j,s}$ are less than 4.5%, as shown in Supplementary Fig. 7. The Zpa applied to each qubit is calibrated based on the mapping relationship

$$Z_\text{sensed} = \tilde{M}_Z \cdot Z_\text{applied}, \tag{7}$$

where Z_sensed and Z_applied are for the Zpas that are sensed by the qubit and applied to the qubit, respectively.

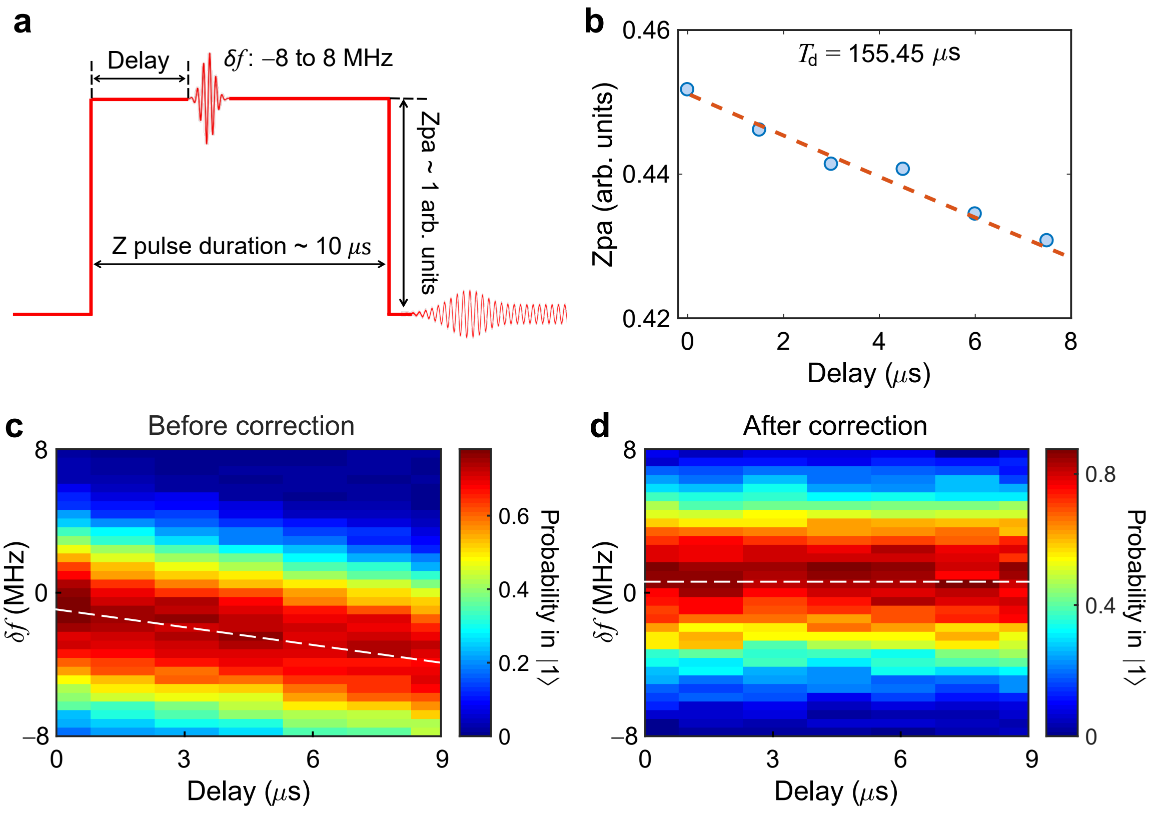

Supplementary Fig. 5. Calibration of the Z pulse distortion for the long-time scale caused by the DC block. (a) Schematic of the calibration pulse sequence. We apply a Z pulse with a fixed duration ($\sim$ 10 μs) and a fixed amplitude ($\sim$ 1 arb. units) and excite the qubit around the target frequency by a X_π pulse. Then, we measure the population of the $|1\rangle$ state for different time delays between the X_π pulse and the Z pulse, given a difference of the frequency δf. (b) An exponential decay function is used to estimate the characteristic decay factor as $T_\mathrm{d} = 155.45$ μs. (c and d) Z pulse shapes obtained by measuring population of $|1\rangle$ before (c) and after (d) the Z correction, respectively.

E. Qubits frequencies

In the 15-qubit experiment, we simulate the two-dimensional (2D) integer quantum Hall effect (QHE) using 15 qubits on one leg of the qubit ladder by tuning the qubits frequencies as $\omega_j(\phi) = \omega_0 + \Delta\sum_{j=1}^{15}\cos(2\pi bj + \phi)$, with the reference frequency being fixed at $\omega_0/2\pi = 4.7$ GHz. Here, ϕ is varied from 0 to 2π in order to obtain various instances of the 1D Aubry-André-Harper (AAH) chains with different on-site potentials $V_j(\phi) = \omega_j(\phi) - \omega_0$, in the rotating frame with respect to ω_0. Then, we experimentally simulate the 1D topological charge pump, which provides an alternative way to explore the 2D integer QHE, by adiabatically varying ϕ initially from $\phi_0 = \frac{5\pi}{3}$ with $\Delta/2\pi = 36$ MHz. In the 30-qubit experiment, we tune the qubits frequencies as $\omega'_{j,s}(\phi) = \omega'_0 + \Delta_s\sum_{j=1}^{15}\cos(2\pi bj + \phi)$, with the reference frequency being fixed at $\omega'_0/2\pi = 4.5$ GHz.

Thus, it is very significant to precisely adjust the qubits frequencies in our experiments. For the single-qubit experiment, it is simple to tune the qubit to the target frequency with a determined relationship between the Zpa and the qubit frequency, as shown in Supplementary Fig. 8a. However, for the multi-qubit case, the target qubits frequencies will shift slightly when other qubits Z signals are applied simultaneously, even though the Z crosstalks have been calibrated by using the Z-crosstalk matrix $\tilde{M}_Z$. This indicates the existence of residual Z crosstalks. Although we can obtain a larger Z-crosstalk matrix $\tilde{M}'_Z$ when other qubits Z pulses are applied, it is not very worthwhile, because to calibrate all the matrix elements are very time consuming.

Here, we perform direct Rabi oscillation measurements on each stand-alone qubit around the reference frequency [from $(\omega_0/2\pi - 36$ MHz) to $(\omega_0/2\pi + 36$ MHz)]. As shown in Supplementary Fig. 8b, to calibrate the Zpa for detuning $Q_{1,\uparrow}$ to 4.7 GHz, we design two Zpa configurations for other qubits to cancel the effect of the residual Z crosstalks. Given the experimental on-site potentials and the coupling strengths between qubits as shown in Supplementary Fig. 3a, the nearest-neighbour (NN) qubit ($Q_{2,\uparrow}$), the next-nearest-neighbour (NNN) qubit ($Q_{3,\uparrow}$) and other qubits are detuned $\pm$80 MHz, $\pm$30 MHz and $\pm$20 MHz with respect to $\omega_0/2\pi$, respectively. Note that there are also other Zpa configurations in addition to those as shown in Supplementary Fig. 8b, which hardly affect the final result.

We fix the driving magnitude of the local transverse field with a resonant frequency at 4.7 GHz and scan the Zpa around

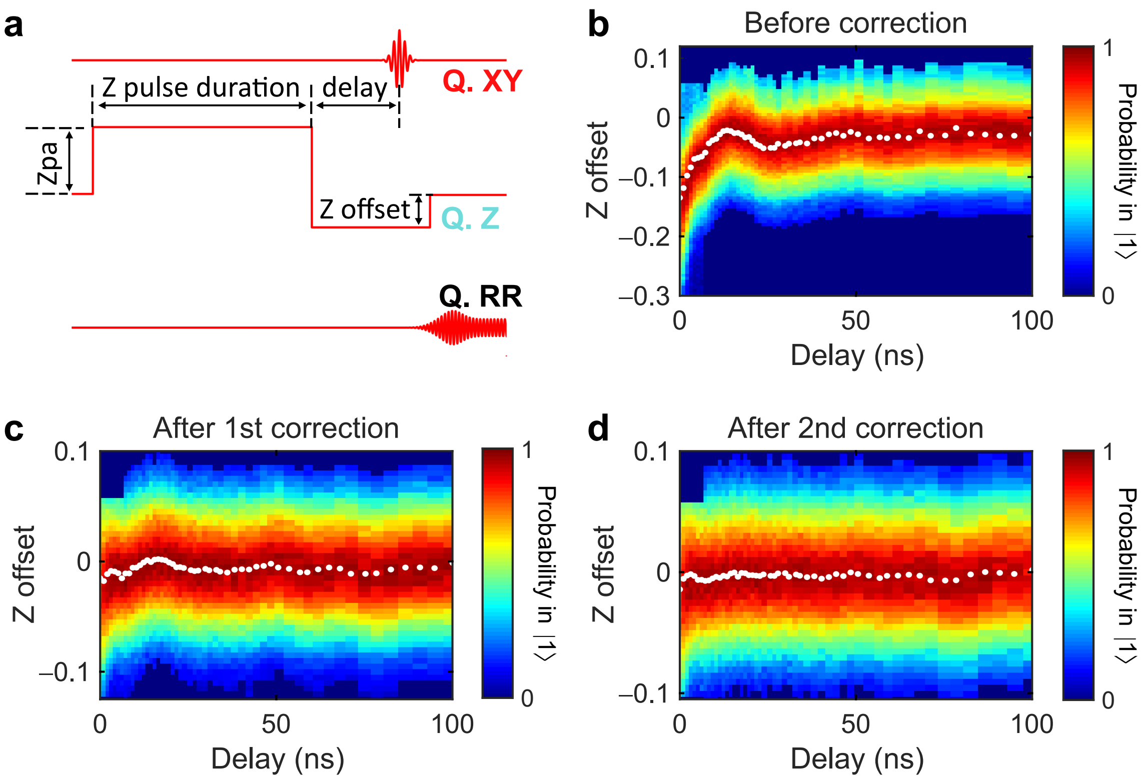

Supplementary Fig. 6. Calibration of the Z pulse distortion for short-time scales. (a) Schematic of the experimental pulse sequence. We apply a Z pulse with a fixed duration ($\sim$ 1 μs) and a fixed amplitude ($\sim$ 1 arb. units) to the qubit for obtaining the shape of the rising (falling) edge by exciting the qubit around the its idle frequency (by varying the Z offset parameter). (b) Pulse shape before the correction. (c and d) Z pulse shapes after the first (c) and second (d) correction procedures, respectively.

the guessed one (marked with a blue star in Supplementary Fig. 8a). For the two Zpa configurations, the 2D Rabi oscillation results are plotted in Supplementary Fig. 8c and 8d, respectively. The slowest Rabi oscillation characterises the optimised Zpa, corresponding to the smallest driving strength as shown in Supplementary Fig. 8e.

Finally, the Zpa used in our experiments (marked with a red star in Supplementary Fig. 8f) is fixed at the average value of the two optimised Zpa configurations. Note that the final Zpa indicates a frequency shift of about 2.5 MHz with respect to that shown in Supplementary Fig. 8a. For simplicity, we can select some typical frequencies and repeat the above optimisation procedure for obtaining a new relationship between the Zpa and qubits frequencies in the region studied (see Supplementary Fig. 8f).

F. Phase calibration

As shown in Fig. 1d in the main text for the pulse sequences for the band structure spectroscopy, a $Y_{\frac{\pi}{2}}$ rotation pulse is applied on the target qubit for measuring the time evolutions of $\langle\hat{\sigma}^x(t)\rangle$ and $\langle\hat{\sigma}^y(t)\rangle$. Before the readout of $Q_{j,s}$ at its idle point in the bases of $\hat{\sigma}^x$ and $\hat{\sigma}^y$, we detune all qubits to their corresponding frequencies for the quench evolution with a time t by applying a Z pulse to each qubit. This operation will accumulate a dynamical phase that needs to be compensated when applying the rotation pulse after the time evolution. For each qubit $Q_{j,s}$, the dynamical phase accumulated during the evolution can be calculated by $\delta\omega_{j,s}t$, where $\delta\omega_{j,s} \equiv \omega_{j,s} - \omega_0$, and $\omega_0/2\pi$ is the target frequency of $Q_{j,s}$ for the quench dynamics. Although the Z pulse distortion has been carefully calibrated before the experiment, there are still some imperfect factors, leading to an additional phase shift, as compared with the theoretical calculations.

Therefore, we design a pulse sequence for the phase calibration. First, a $Y_{\frac{\pi}{2}}$ pulse is applied to $Q_{j,s}$, and then, all qubits frequencies are arranged with two configurations, similar as the case in Supplementary Fig. 8b for the calibration of the qubits frequencies. Then, we apply another $Y_{\frac{\pi}{2}}$ pulse to $Q_{j,s}$ and vary its microwave phase for obtaining the probability when Q_j is in $|1\rangle$ as a function of t and the phase difference $(\varphi - \delta\omega_{j,s}t)$. For these two Zpa configurations, we obtain the experimental results shown in Supplementary Fig. 9a and 9b, respectively. To calculate the phase compensation for the second rotation pulse, we perform a standard cosine fitting to the probability of a qubit $Q_{j,s}$ in the $|1\rangle$ state, $P_{j,s}$, as a function of $(\varphi - \delta\omega_{j,s}t)$ for each evolution time t. The results are shown in Supplementary Fig. 9c, and the final phase compensation can be obtained as $(\varphi_1 + \varphi_2)/2$, where φ_1 and φ_2

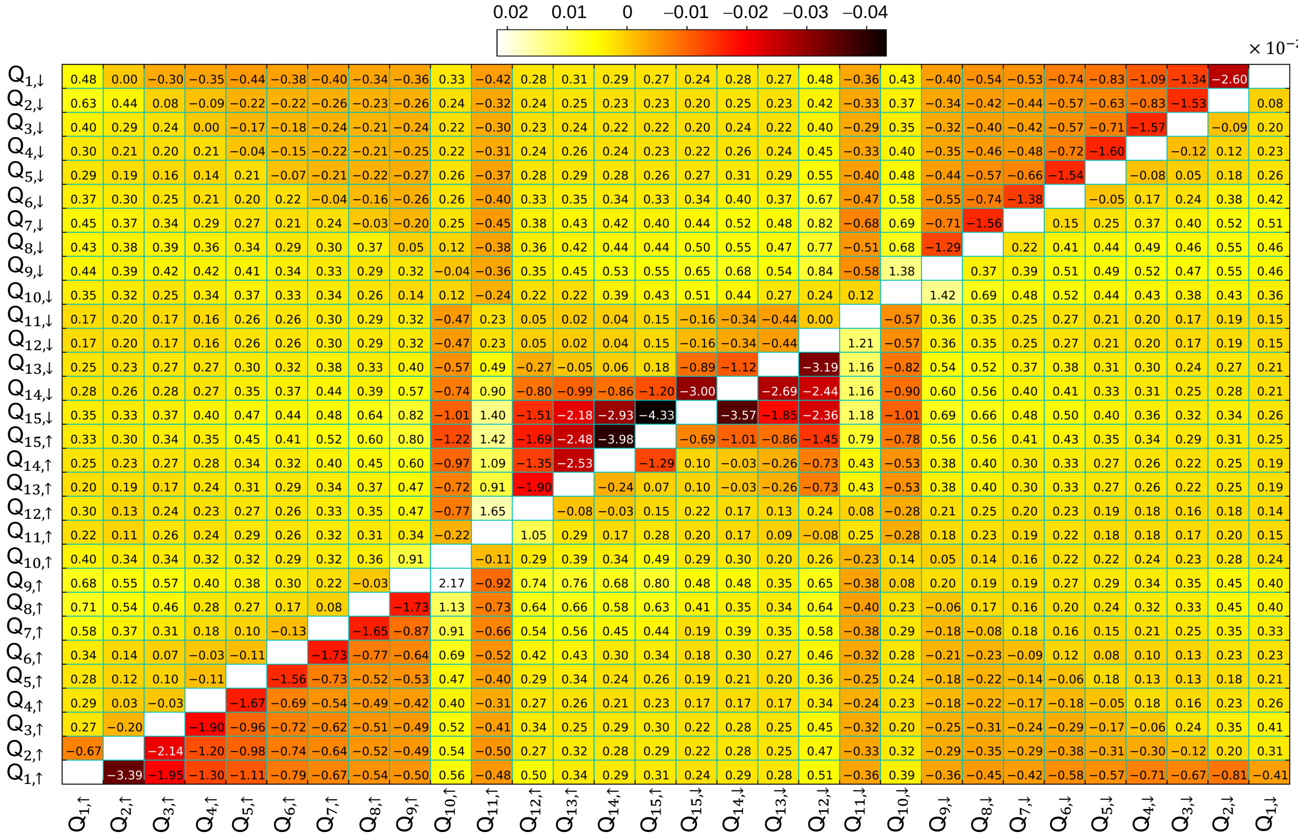

Supplementary Fig. 7. Z crosstalk. Z crosstalk matrix $\tilde{M}_Z$, of which each element $(\tilde{M}_Z)_{i,r;j,s}$ denotes the sensed Zpa by $Q_{j,s}$ when a unity Zpa (1 arb. units) is applied to $Q_{i,r}$.

are the phase compensations under two Zpa configurations, respectively. After the above phase calibration process and adding the phase compensation on each qubit, we obtain the experimental results shown in Supplementary Fig. 9d, which indicate that the phase shift is compensated for all evolution times.

Supplementary Note 3. SUPPLEMENTARY EXPERIMENTAL DATA

A. Single-excitation quantum walks

After initialising the system with a state $|0\rangle^{\otimes N}$, we excite one qubit $Q_{j,s}$ with a X_π pulse, tune all qubits to their corresponding frequencies and measure them at a time t after their free evolutions. The total repeat count for the measurement at each time on each qubit is 3,000.

In the 15-qubit experiment, for $b = \frac{1}{3}$ and different values of $\Delta_\uparrow/2\pi$ and ϕ, the experimental results of the time evolution of the excitation probability $P_{j,\uparrow}$ are shown in Supplementary Fig. 10, 11, 12 and 13 for:

(i) $\Delta_\uparrow/2\pi = 0$ MHz,

(ii) $\Delta_\uparrow/2\pi = 12$ MHz with $\phi = \frac{2\pi}{3}$,

(iii) $\Delta_\uparrow/2\pi = 12$ MHz with $\phi = \frac{\pi}{10}$,

(iv) $\Delta_\uparrow/2\pi = 12$ MHz with $\phi = \pi$,

respectively, which are compared with the theoretical predictions using the parameters in Supplementary Table 1.

In the 30-qubit experiment, for $b = \frac{1}{3}$ and different values of $\Delta_s/2\pi$ and ϕ, the experimental results are shown in Supplementary Fig. 14, 15, 16, 17, and 18 for:

(i) $\Delta_\uparrow/2\pi = \Delta_\downarrow/2\pi = 0$ MHz,

(ii) $\Delta_\uparrow/2\pi = \Delta_\downarrow/2\pi = 12$ MHz with $\phi = \frac{2\pi}{3}$,

(iii) $\Delta_\uparrow/2\pi = -\Delta_\downarrow/2\pi = 12$ MHz with $\phi = \frac{2\pi}{3}$,

(iv) $\Delta_\uparrow/2\pi = \Delta_\downarrow/2\pi = 12$ MHz with $\phi = \frac{\pi}{10}$,

(v) $\Delta_\uparrow/2\pi = -\Delta_\downarrow/2\pi = 12$ MHz with $\phi = \frac{\pi}{10}$,

respectively, which are compared with the theoretical predictions using the parameters in Supplementary Table 2.

To evaluate the performance of the experimental results of the quantum walks, we calculate the fidelity of the evolved

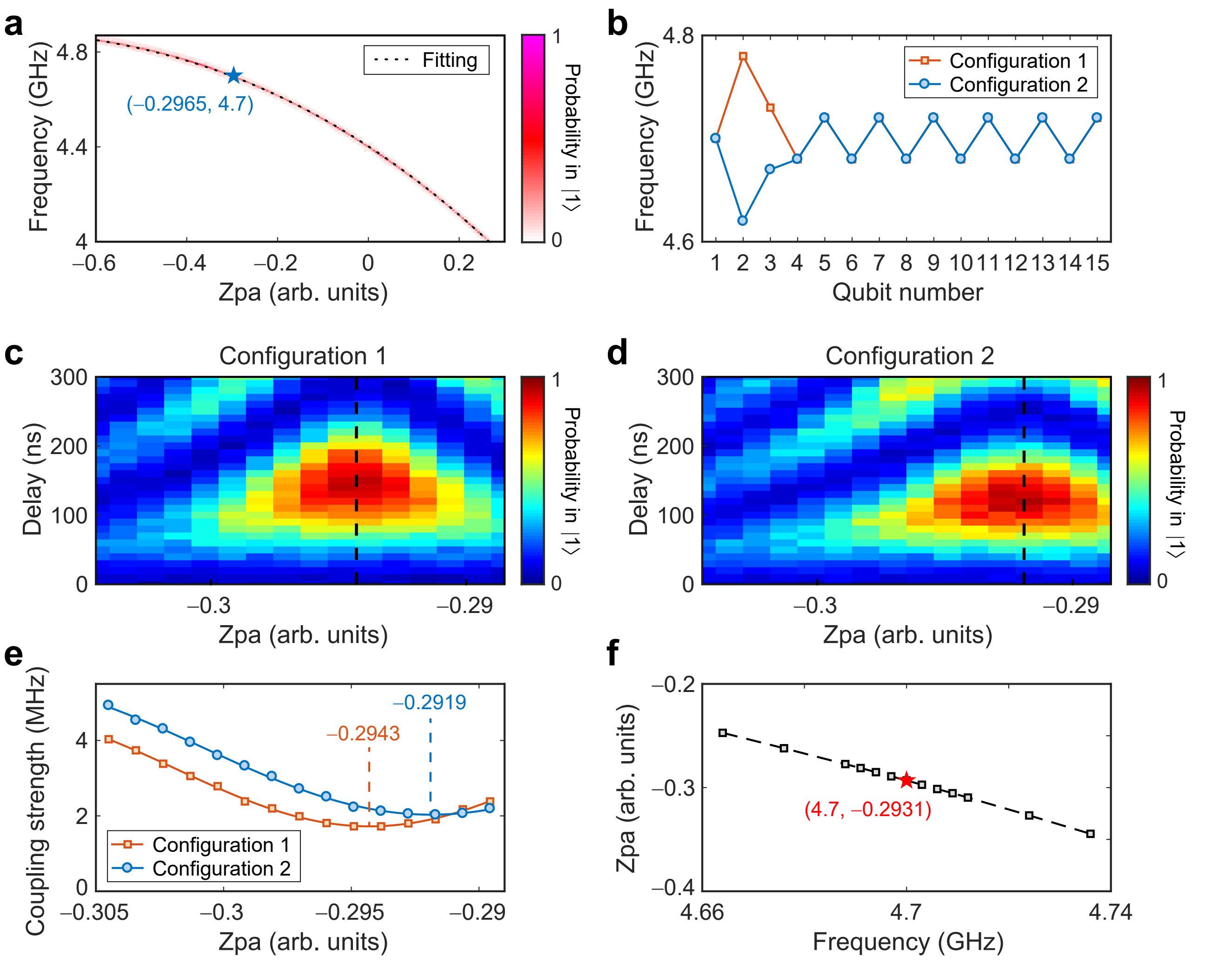

Supplementary Fig. 8. Calibration of qubits frequencies by using the Rabi oscillation measurements. (a) Relationship between the Zpa and the qubits frequencies. The experimental data are extracted from the automatic spectroscopy measurement. (b) Two Zpa configurations for adjusting $Q_{1,\uparrow}$ to 4.7 GHz by using the Rabi oscillation measurements. (c and d) Experimental Rabi oscillation results under two Zpa configurations, respectively. (e) The calculated driving strengths for two Zpa configurations. The smallest driving strength corresponds to the optimal Zpa. (f) The updated relationship between the Zpa and the qubits frequencies in the region studied. The red star is obtained by averaging the two optimal Zpas when using different Zpa configurations.

state using

$$F(t) = \sum_{j,s} \sqrt{p_{j,s}(t) q_{j,s}(t)}, \tag{8}$$

where $p_{j,s}(t)$ and $q_{j,s}(t)$ are the experimental and theoretical probability distributions of the measurements on $Q_{j,s}$ in the $|1\rangle$ state. We consider the mean fidelity that is averaged for different initial states with the excitation on different qubits. As shown in Supplementary Fig. 19, the relatively high fidelity (above around 0.9 and 0.85 within 500 ns for the 15-qubit and 30-qubit experiments, respectively) for all on-site potentials configurations imply that our experimental results are well consistent with the theoretical simulations.

B. Band structure spectroscopy

After initialising the selected qubits to their idle points, we place one target qubit $Q_{j,s}$ in the superposed state $|+_{j,s}\rangle = (|0_{j,s}\rangle + |1_{j,s}\rangle)/\sqrt{2}$, using a $Y_{\frac{\pi}{2}}$ pulse. Then, we detune all selected qubits to their corresponding frequencies for the quench dynamics with a time t. Then, we measure $Q_{j,s}$ at its idle point in the $\hat{\sigma}^x$ and $\hat{\sigma}^y$ bases, and the total repeat count for each measurement on each qubit is 2,400. For each ϕ, the time evolutions of $\hat{\sigma}^x_{j,s}$ and $\hat{\sigma}^y_{j,s}$ are recorded. We then calculate the squared Fourier transformation (FT) magnitude $|A_{j,s}|^2$ of the response function $\chi_{j,s}(t) \equiv \langle\hat{\sigma}^x_{j,s}(t)\rangle + i\langle\hat{\sigma}^y_{j,s}(t)\rangle$ for each qubit.

In the 15-qubit experiment, for $b = \frac{1}{3}$ and different values of $\Delta_{\uparrow}/2\pi$ and ϕ, the experimental results of the normalised

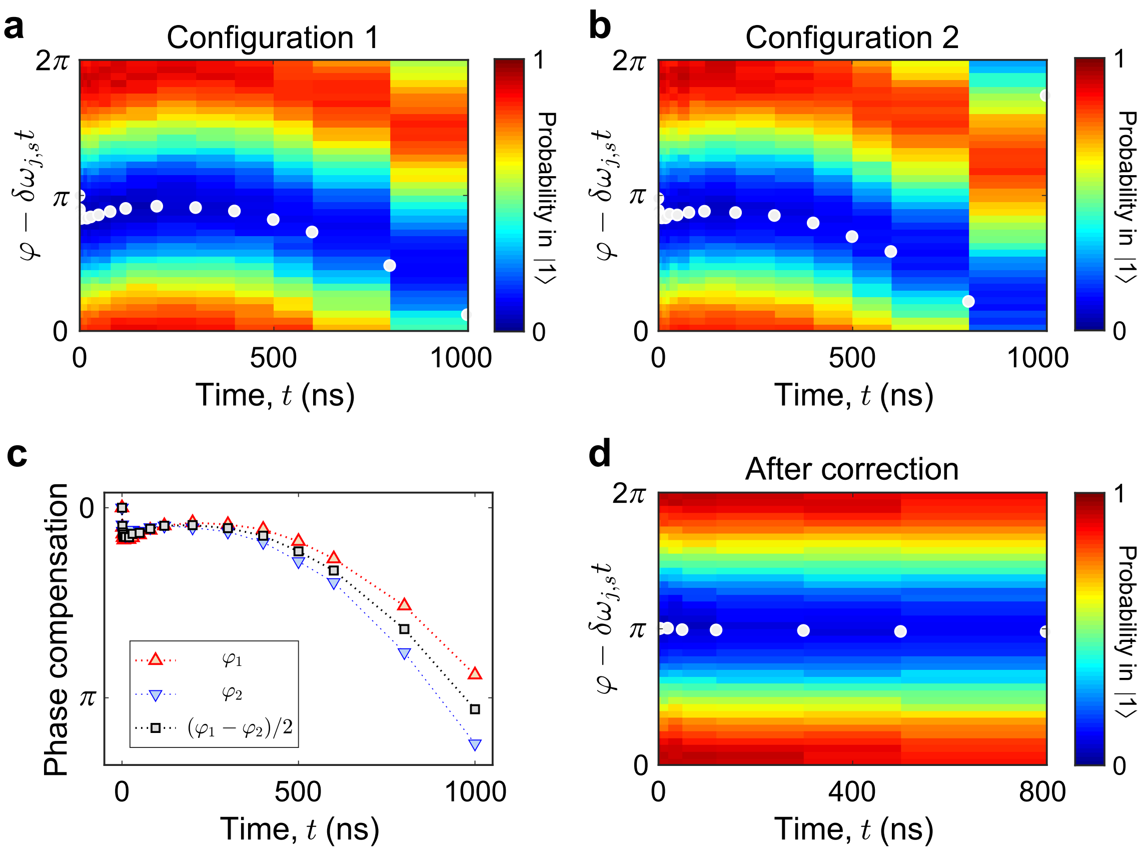

Supplementary Fig. 9. Phase calibration. (a and b) Time evolutions of the probability of a qubit $Q_{j,s}$ in the $|1\rangle$ state for two Zpa configurations, respectively. (c) The phase shift obtained by using the standard cosine fitting for two Zpa configurations. The final phase compensation can be obtained as $(\varphi_1 + \varphi_2)/2$, where φ_1 and φ_2 are the phase compensations under two Zpa configurations, respectively. (d) Time evolution of the probability of $Q_{j,s}$ in the $|1\rangle$ state after the phase calibration.

squared FT magnitude $|A_{j,\uparrow}|^2_{\text{n.m.}}$ for each selected target qubit are shown in Supplementary Fig. 21 and 22 for $\Delta_\uparrow/2\pi = 0$ MHz and $\Delta_\uparrow/2\pi = 12$ MHz, respectively.

In the 30-qubit experiment, for $b = \frac{1}{3}$ and different values of $\Delta_s/2\pi$ and ϕ, the experimental results of the normalised squared FT magnitude $|A_{j,s}|^2_{\text{n.m.}}$ for each selected target qubit are shown in Supplementary Fig. 23 and 24 for $\Delta_\uparrow/2\pi = \Delta_\downarrow/2\pi = 12$ MHz and $\Delta_\uparrow/2\pi = -\Delta_\downarrow/2\pi = 12$ MHz, respectively.

For the case $\Delta_\uparrow/2\pi = \Delta_\downarrow/2\pi = 12$ MHz, we choose 15 qubits for the band structure measurements, which include all four corner qubits, i.e., $Q_{1,\uparrow}$, $Q_{1,\downarrow}$, $Q_{15,\uparrow}$, and $Q_{15,\downarrow}$, and 11 bulk qubits, i.e., $Q_{2,\uparrow}$, $Q_{4,\uparrow}$, $Q_{5,\uparrow}$, $Q_{6,\uparrow}$, $Q_{7,\uparrow}$, $Q_{8,\uparrow}$, $Q_{10,\uparrow}$, $Q_{13,\uparrow}$, $Q_{14,\uparrow}$, $Q_{12,\downarrow}$, and $Q_{3,\downarrow}$.

For the case $\Delta_\uparrow/2\pi = -\Delta_\downarrow/2\pi = 12$ MHz, we choose 20 qubits for the band structure measurements, which include all four corner qubits, i.e., $Q_{1,\uparrow}$, $Q_{1,\downarrow}$, $Q_{15,\uparrow}$, and $Q_{15,\downarrow}$, and 16 bulk qubits, i.e., $Q_{2,\uparrow}$, $Q_{4,\uparrow}$, $Q_{5,\uparrow}$, $Q_{6,\uparrow}$, $Q_{8,\uparrow}$, $Q_{10,\uparrow}$, $Q_{12,\uparrow}$, $Q_{14,\downarrow}$, $Q_{12,\downarrow}$, $Q_{10,\downarrow}$, $Q_{9,\downarrow}$, $Q_{8,\downarrow}$, $Q_{6,\downarrow}$, $Q_{5,\downarrow}$, $Q_{4,\downarrow}$, and $Q_{3,\downarrow}$.

Then, we compare in Supplementary Fig. 20 the experimentally measured band structures by summing the FT magnitudes of all selected qubits with the theoretical predictions using the experimental parameters in Supplementary Fig. 3a and 3b for the 15-qubit and 30-qubit experiments, respectively. The average deviations of the peaks of the normalised FT signals $I_\phi^{\text{n.m.}}$ (red crosses) from the theoretical band structures (black dashed curves) are 0.247 MHz, 0.264 MHz, 0.568 MHz, and 0.512 MHz for:

(*i*) 15-qubit experiment with $\Delta_\uparrow/2\pi = 0$ MHz,

(*ii*) 15-qubit experiment with $\Delta_\uparrow/2\pi = 12$ MHz and $b = \frac{1}{3}$,

(*iii*) 30-qubit experiment with $\Delta_\uparrow/2\pi = \Delta_\downarrow/2\pi = 12$ MHz and $b = \frac{1}{3}$,

(*iv*) 30-qubit experiment with $\Delta_\uparrow/2\pi = -\Delta_\downarrow/2\pi = 12$ MHz and $b = \frac{1}{3}$,

respectively. Compared with the ranges of the eigenenergies -20 to 20 MHz and -30 to 30 MHz for the 15-qubit and 30-qubit systems, respectively, the small average deviations imply that our band structure spectroscopic measurements are very accurate.

C. Quantum charge pumping and fast pumping

In addition to the topological charge pumping data as shown in Fig. 3c1–3c3 and 3d in the main text, we have also performed experiments with a faster pumping rate, which clearly demonstrate fast pumping. In details, we have decreased the period T from 1,100 ns to 410 ns, corresponding to a faster ramping speed of ϕ. The experimental results for the displacement of the centre of mass (CoM) δx versus the time t/T are compared with numerical simulations in Supplementary Fig. 25a. It is obvious that the pumping process with a period $T = 410$ ns shows fast pumping with a large derivation from the Chern number $+1$. The experimental data of the evolutions of distributions $P_j(t)$ during the topological pumping and the displacement of the CoM with a period $T = 410$ ns are compared with the data with $T = 1{,}100$ ns in Supplementary Fig. 26. For fast pumping with $T = 410$ ns, we maintained a fixed sample of 3,000 single-shot readouts and repeated the measurement procedure 8 times (16 times for the no pump case) for estimating the mean values and standard deviations at each evolution time t. Furthermore, Supplementary Fig. 25b shows the numerical results of the displacement of the CoM in one pumping cycle with different periods, which indicates a fast pumping region in our system when $T \lesssim 610$ ns.

D. Numerical details

Numerical simulations are performed using the QuTiP [10, 11] (the quantum toolbox in Python) and NumPy. The time evolutions of the systems are numerically simulated using QuTiPs master equation solver mesolve, where the parameters in Supplementary Fig. 3 are used. Because the evolution time is much shorter than the qubits' decoherence times $t \ll \bar{T}^1, T^{2*}$, we do not consider the effect of decoherence in simulations.

[1] Z. Chen, A. Megrant, J. Kelly, R. Barends, J. Bochmann, Y. Chen, B. Chiaro, A. Dunsworth, E. Jeffrey, J. Y. Mutus, P. J. J. O'Malley, C. Neill, P. Roushan, D. Sank, A. Vainsencher, J. Wenner, T. C. White, A. N. Cleland, and J. M. Martinis, Fabrication and characterization of aluminum airbridges for superconducting microwave circuits, Appl. Phys. Lett. **104**, 052602 (2014).

[2] P. Roushan, C. Neill, J. Tangpanitanon, V. M. Bastidas, A. Megrant, R. Barends, Y. Chen, Z. Chen, B. Chiaro, A. Dunsworth, A. Fowler, B. Foxen, M. Giustina, E. Jeffrey, J. Kelly, E. Lucero, J. Mutus, M. Neeley, C. Quintana, D. Sank, A. Vainsencher, J. Wenner, T. White, H. Neven, D. G. Angelakis, and J. Martinis, Spectroscopic signatures of localization with interacting photons in superconducting qubits, Science **358**, 1175 (2017).

[3] Y. Ye, Z.-Y. Ge, Y. Wu, S. Wang, M. Gong, Y.-R. Zhang, Q. Zhu, R. Yang, S. Li, F. Liang, J. Lin, Y. Xu, C. Guo, L. Sun, C. Cheng, N. Ma, Z. Y. Meng, H. Deng, H. Rong, C.-Y. Lu, C.-Z. Peng, H. Fan, X. Zhu, and J.-W. Pan, Propagation and localization of collective excitations on a 24-qubit superconducting processor, Phys. Rev. Lett. **123**, 050502 (2019).

[4] Z. Yan, Y.-R. Zhang, M. Gong, Y. Wu, Y. Zheng, S. Li, C. Wang, F. Liang, J. Lin, Y. Xu, C. Guo, L. Sun, C.-Z. Peng, K. Xia, H. Deng, H. Rong, J. Q. You, F. Nori, H. Fan, X. Zhu, and J.-W. Pan, Strongly correlated quantum walks with a 12-qubit superconducting processor, Science **364**, 753 (2019).

[5] F. Motzoi, J. M. Gambetta, P. Rebentrost, and F. K. Wilhelm, Simple pulses for elimination of leakage in weakly nonlinear qubits, Phys. Rev. Lett. **103**, 110501 (2009).

[6] Y. Zheng, C. Song, M.-C. Chen, B. Xia, W. Liu, Q. Guo, L. Zhang, D. Xu, H. Deng, K. Huang, Y. Wu, Z. Yan, D. Zheng, L. Lu, J.-W. Pan, H. Wang, C.-Y. Lu, and X. Zhu, Solving systems of linear equations with a superconducting quantum processor, Phys. Rev. Lett. **118**, 210504 (2017).

[7] C. Neill, P. Roushan, K. Kechedzhi, S. Boixo, V. Isakov S., V. Smelyanskiy, A. Megrant, B. Chiaro, A. Dunsworth, K. Arya, R. Barends, B. Burkett, Y. Chen, Z. Chen, A. Fowler, B. Foxen, M. Giustina, R. Graff, E. Jeffrey, T. Huang, J. Kelly, P. Klimov, E. Lucero, J. Mutus, M. Neeley, C. Quintana, D. Sank, A. Vainsencher, J. Wenner, T. C. White, H. Neven, and J. M. Martinis, A blueprint for demonstrating quantum supremacy with superconducting qubits, Science **360**, 195 (2018).

[8] M. A. Rol, L. Ciorciaro, F. K. Malinowski, B. M. Tarasinski, R. E. Sagastizabal, C. C. Bultink, Y. Salathe, N. Haandbaek, J. Sedivy, and L. DiCarlo, Time-domain characterization and correction of on-chip distortion of control pulses in a quantum processor, Appl. Phys. Lett. **116**, 054001 (2020).

[9] C. Song, K. Xu, W. X. Liu, C. P. Yang, S. B. Zheng, H. Deng, Q. W. Xie, K. Q. Huang, Q. J. Guo, L. B. Zhang, P. F. Zhang, D. Xu, D. N. Zheng, X. B. Zhu, H. Wang, Y. A. Chen, C. Y. Lu, S. Y. Han, and J. W. Pan, 10-qubit entanglement and parallel logic operations with a superconducting circuit, Phys. Rev. Lett. **119**, 180511 (2017).

[10] J. R. Johansson, P. D. Nation, and F. Nori, Qutip: An open-source Python framework for the dynamics of open quantum systems, Comput. Phys. Commun. **183**, 1760 (2012).

[11] J. R. Johansson, P. D. Nation, and F. Nori, Qutip 2: A Python framework for the dynamics of open quantum systems, Comput. Phys. Commun. **184**, 1234 (2013).

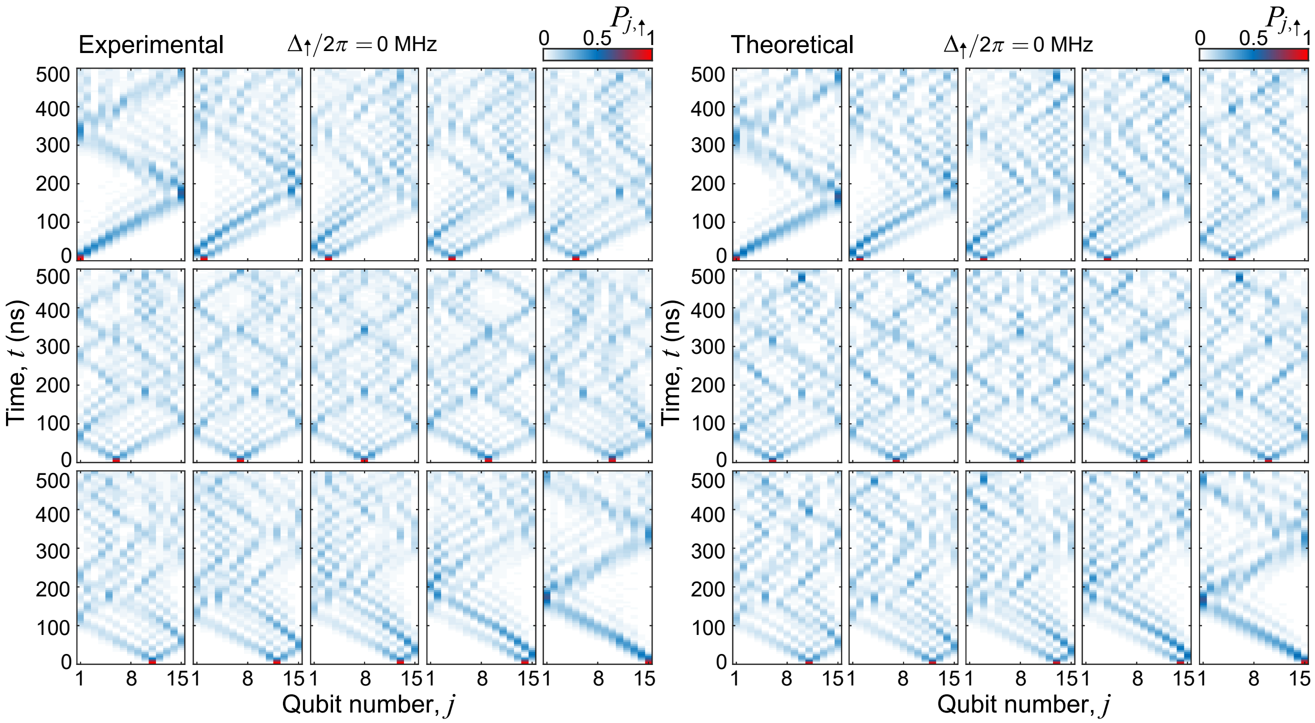

Supplementary Fig. 10. Time evolution of the excitation probability $P_{j,\uparrow}$ with $\Delta_\uparrow/2\pi = 0$ MHz after initially exciting a qubit on the 15-qubit chain.

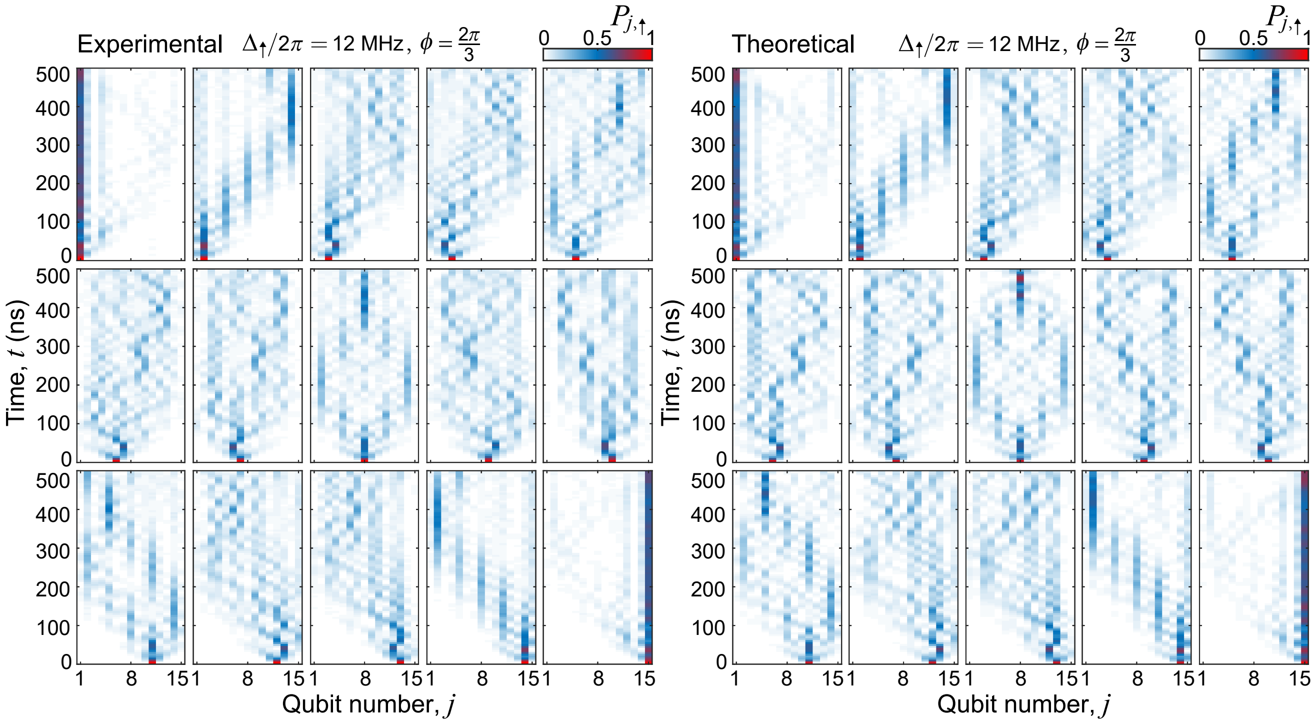

Supplementary Fig. 11. Time evolution of the excitation probability $P_{j,\uparrow}$ with $\Delta_\uparrow/2\pi = 12$ MHz, $b = \frac{1}{3}$, and $\phi = \frac{2\pi}{3}$ after initially exciting a qubit on the 15-qubit chain.

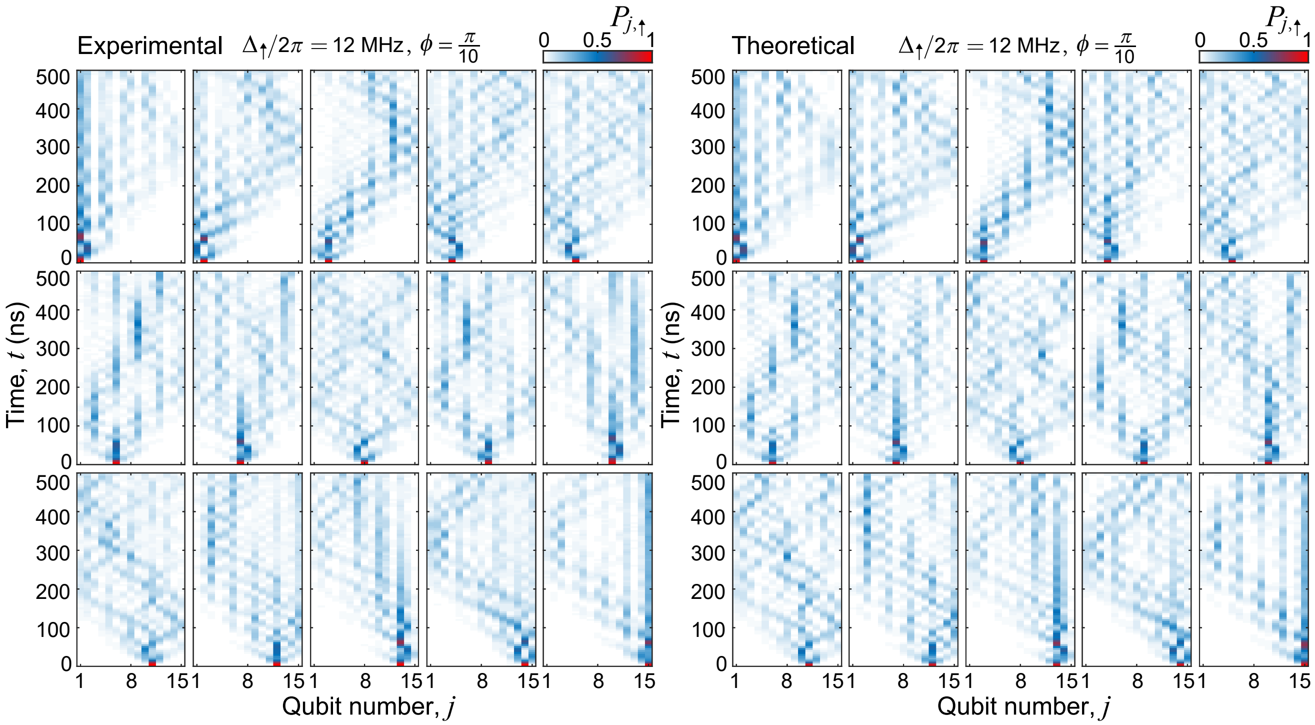

Supplementary Fig. 12. Time evolution of the excitation probability $P_{j,\uparrow}$ with $\Delta_\uparrow/2\pi = 12$ MHz, $b = \frac{1}{3}$, and $\phi = \frac{\pi}{10}$ after initially exciting a qubit on the 15-qubit chain.

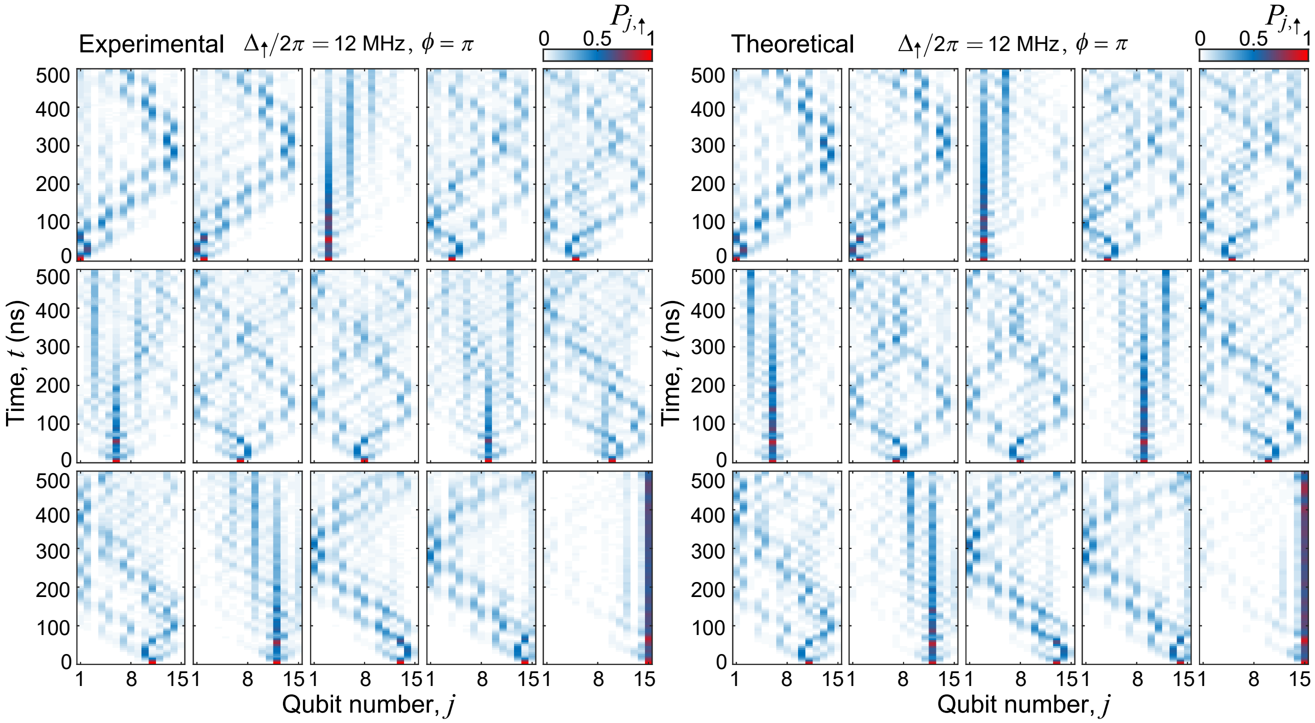

Supplementary Fig. 13. Time evolution of the excitation probability $P_{j,\uparrow}$ with $\Delta_\uparrow/2\pi = 12$ MHz, $b = \frac{1}{3}$, and $\phi = \pi$ after initially exciting each qubit on the 15-qubit chain.

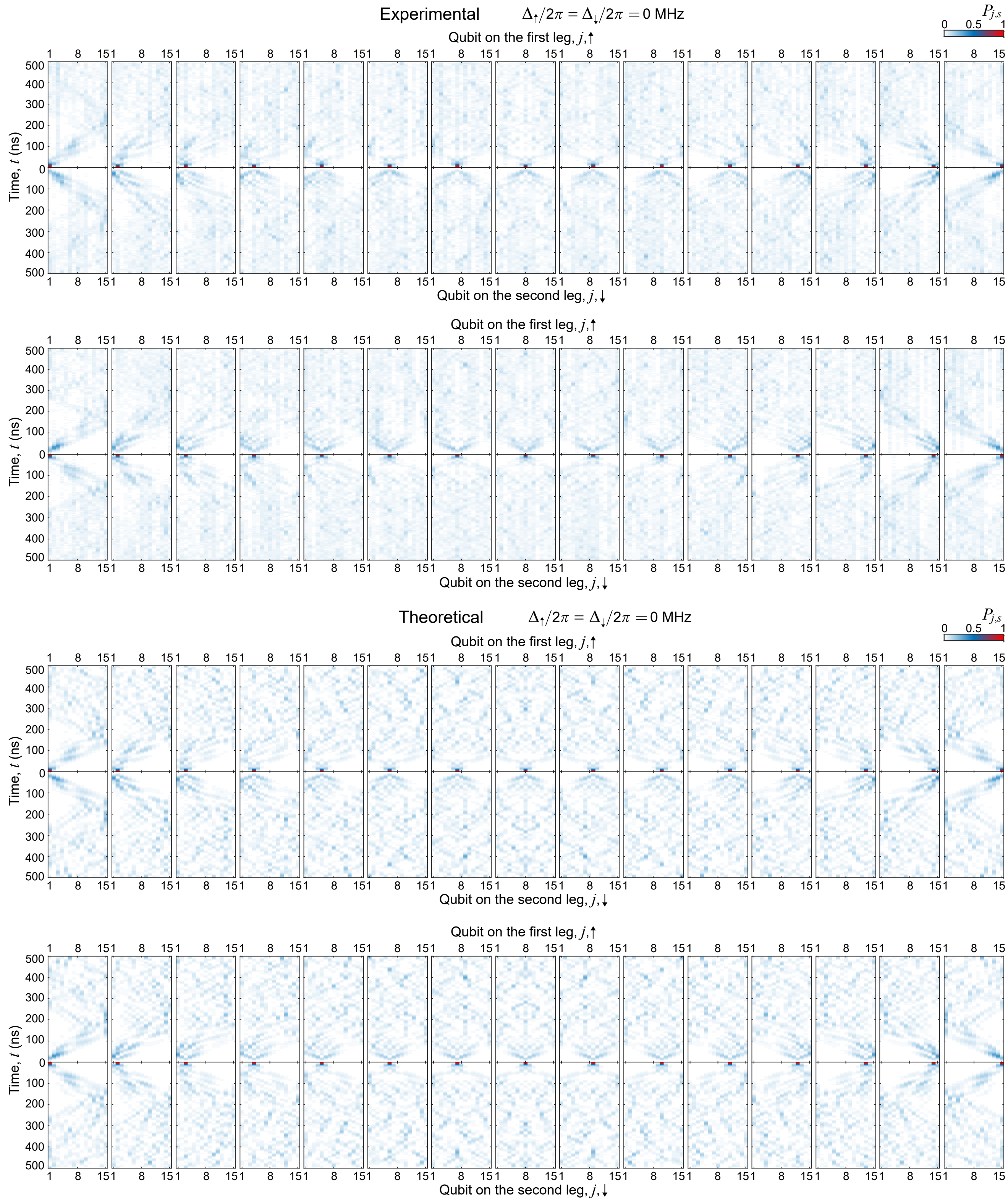

Supplementary Fig. 14. Time evolution of the excitation probability $P_{j,s}$ with $\Delta_\uparrow/2\pi = \Delta_\downarrow/2\pi = 0$ MHz after initially exciting each qubit on the 30-qubit ladder.

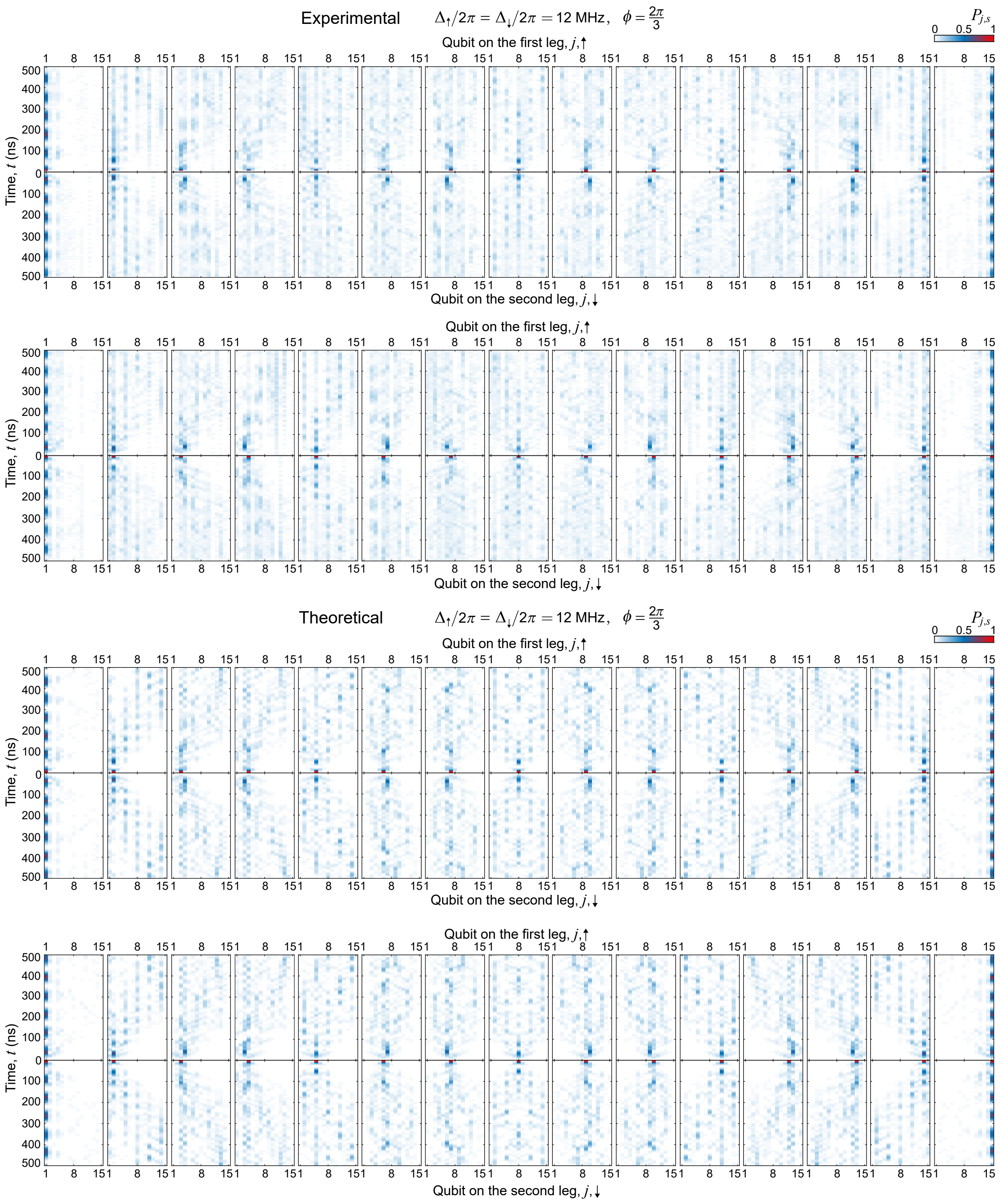

Supplementary Fig. 15. Time evolution of the excitation probability $P_{j,s}$ with $\Delta_\uparrow/2\pi = \Delta_\downarrow/2\pi = 12$ MHz, $b = \frac{1}{3}$, and $\phi = \frac{2\pi}{3}$ after initially exciting each qubit on the 30-qubit ladder.

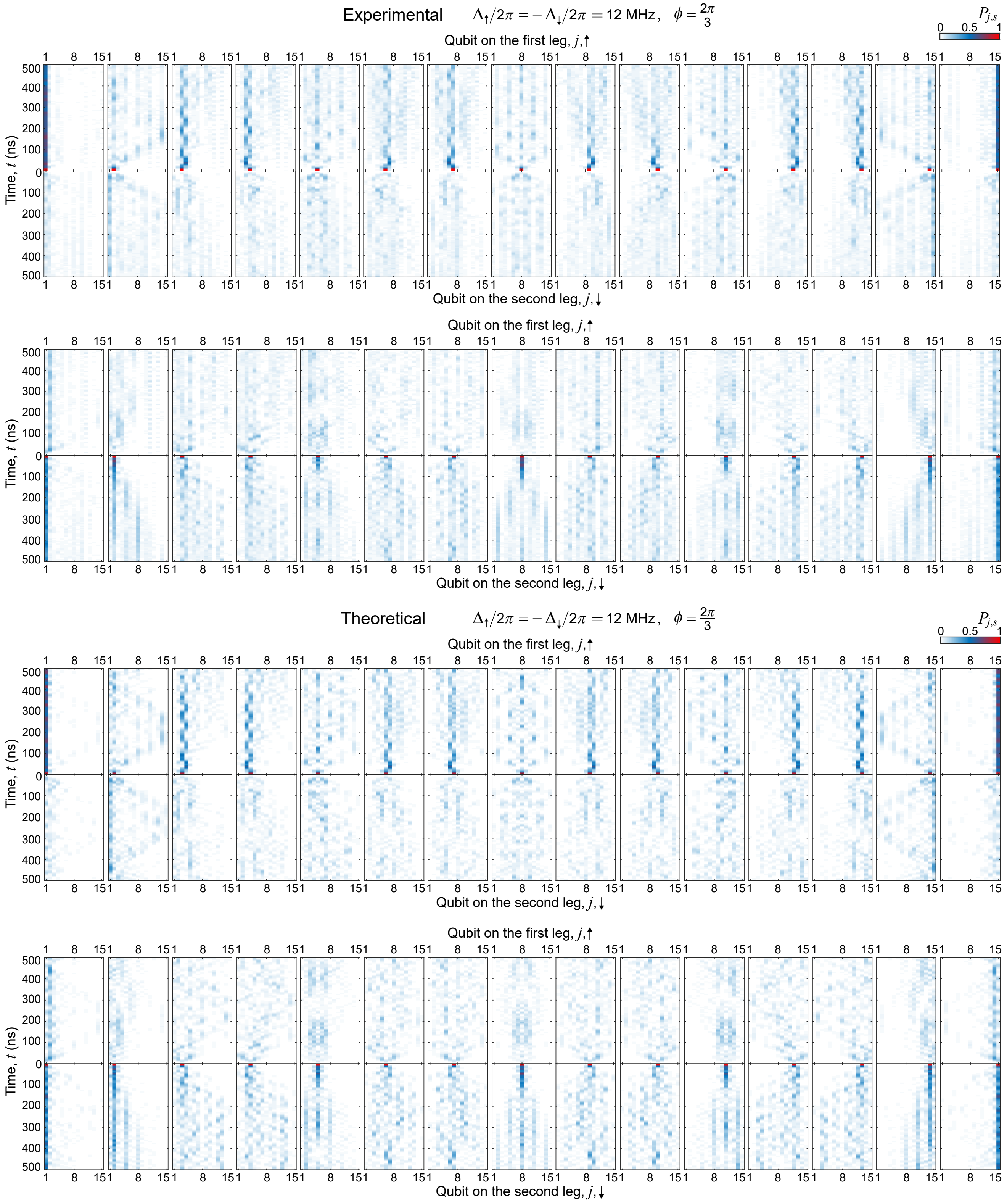

Supplementary Fig. 16. Time evolution of the excitation probability $P_{j,s}$ with $\Delta_\uparrow/2\pi = -\Delta_\downarrow/2\pi = 12$ MHz, $b = \frac{1}{3}$, and $\phi = \frac{2\pi}{3}$ after initially exciting each qubit on the 30-qubit ladder.

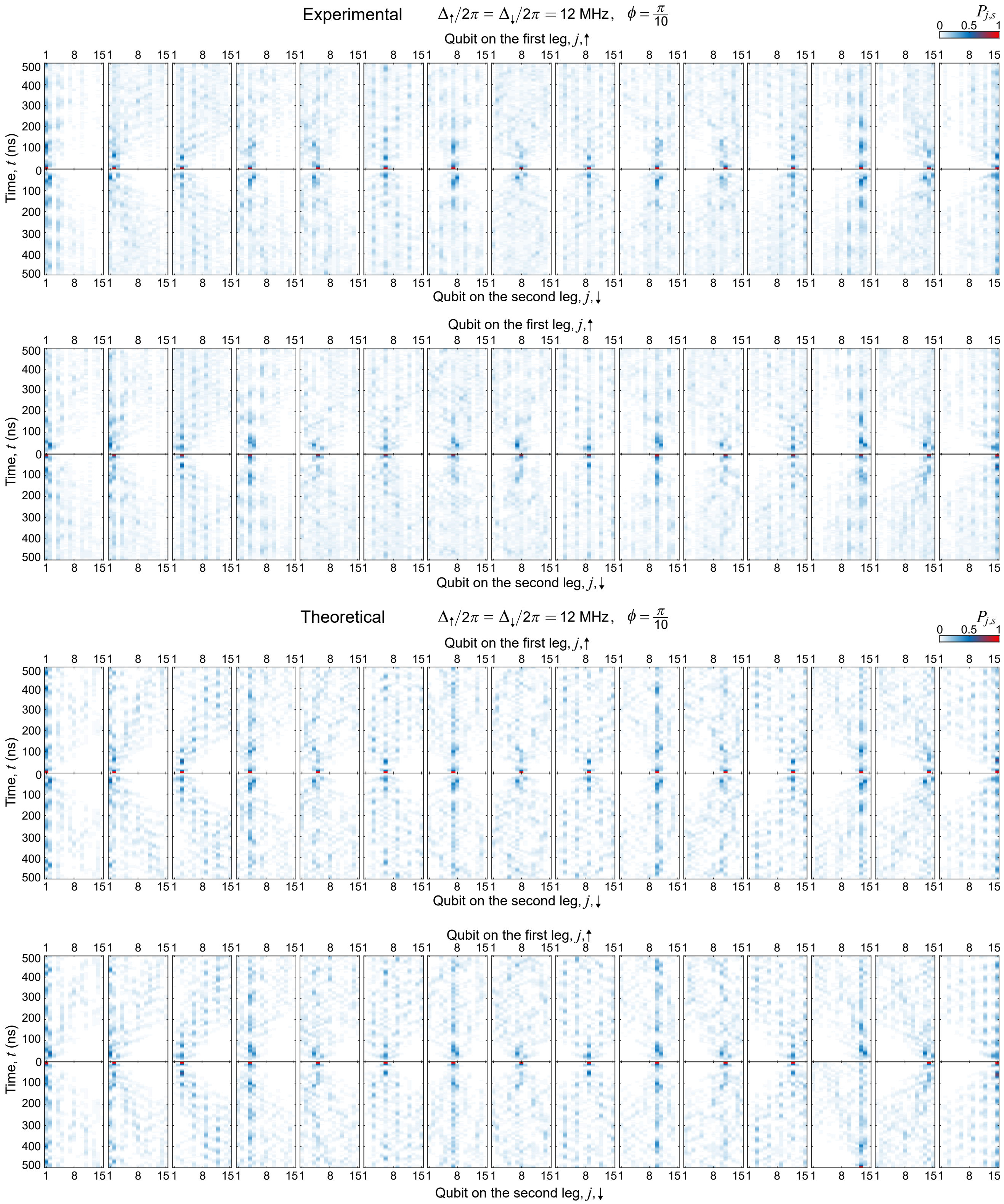

Supplementary Fig. 17. Time evolution of the excitation probability $P_{j,s}$ with $\Delta_\uparrow/2\pi = \Delta_\downarrow/2\pi = 12$ MHz, $b = \frac{1}{3}$, and $\phi = \frac{\pi}{10}$ after initially exciting each qubit on the 30-qubit ladder.

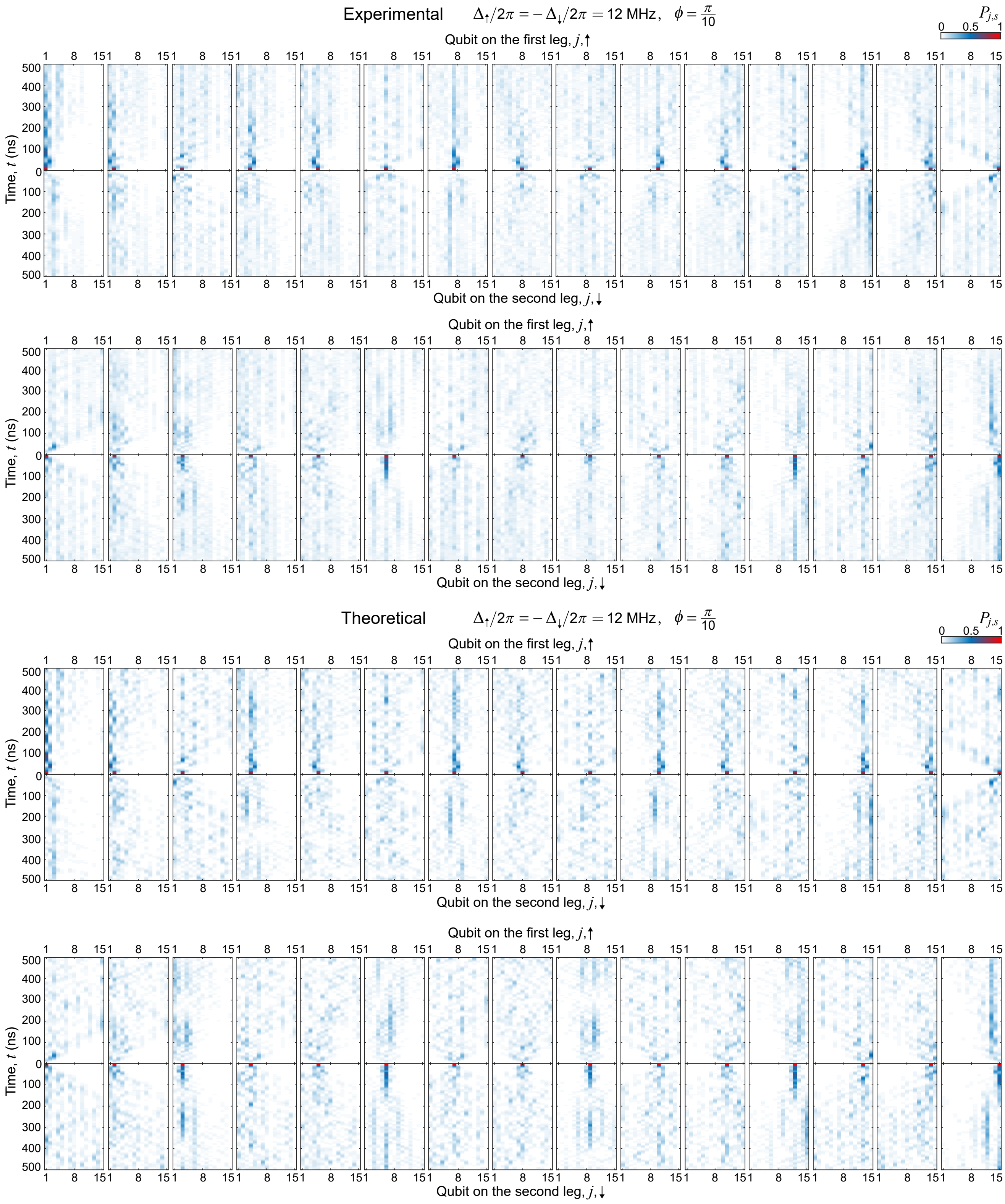

Supplementary Fig. 18. Time evolution of the excitation probability $P_{j,s}$ with $\Delta_\uparrow/2\pi = -\Delta_\downarrow/2\pi = 12$ MHz, $b=\frac{1}{3}$, and $\phi=\frac{\pi}{10}$ after initially exciting each qubit on the 30-qubit ladder.

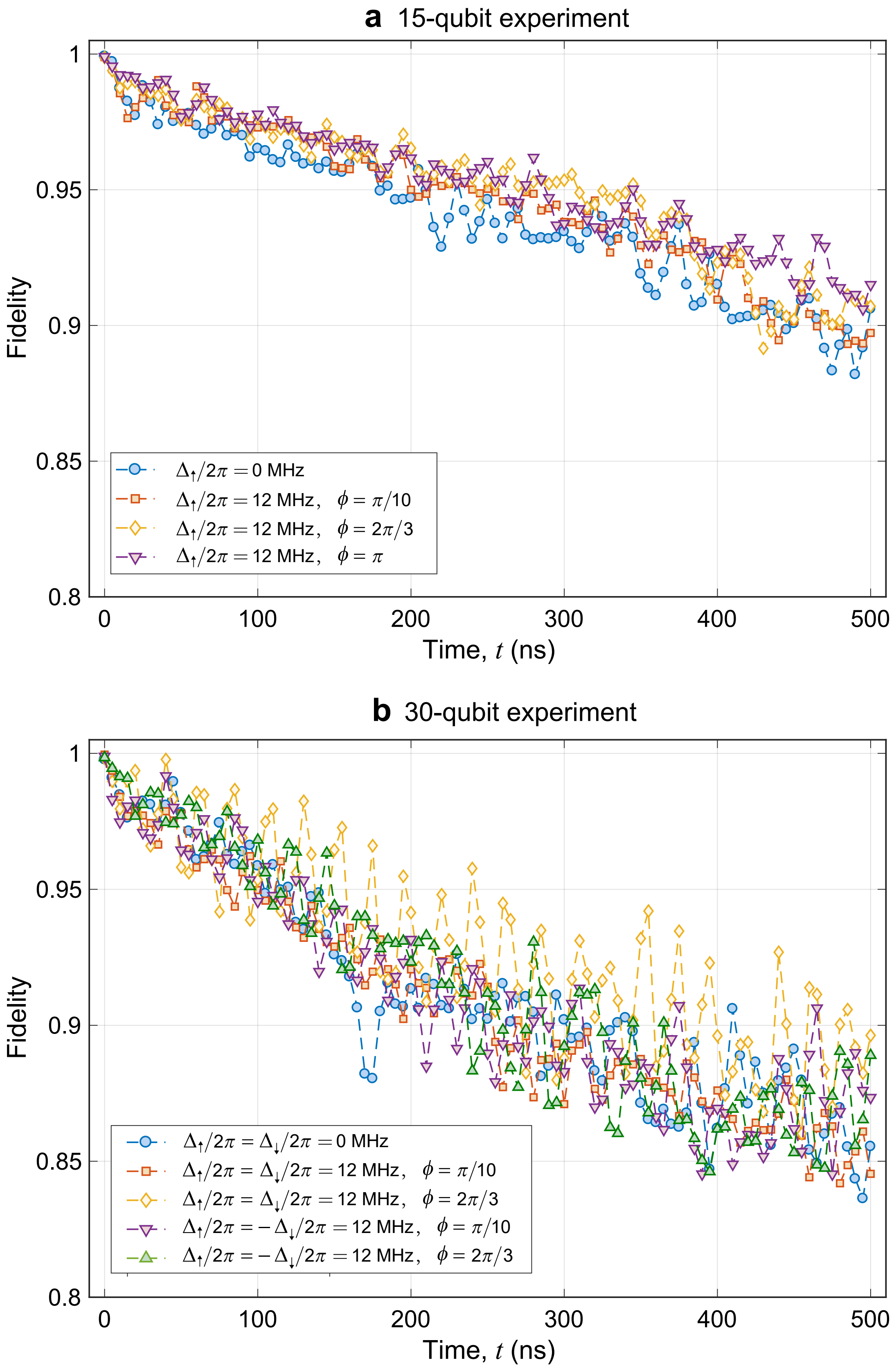

Supplementary Fig. 19. The fidelity for the measured and theoretical probability distributions of the 15-qubit (a) and 30-qubit (b) quantum walks.

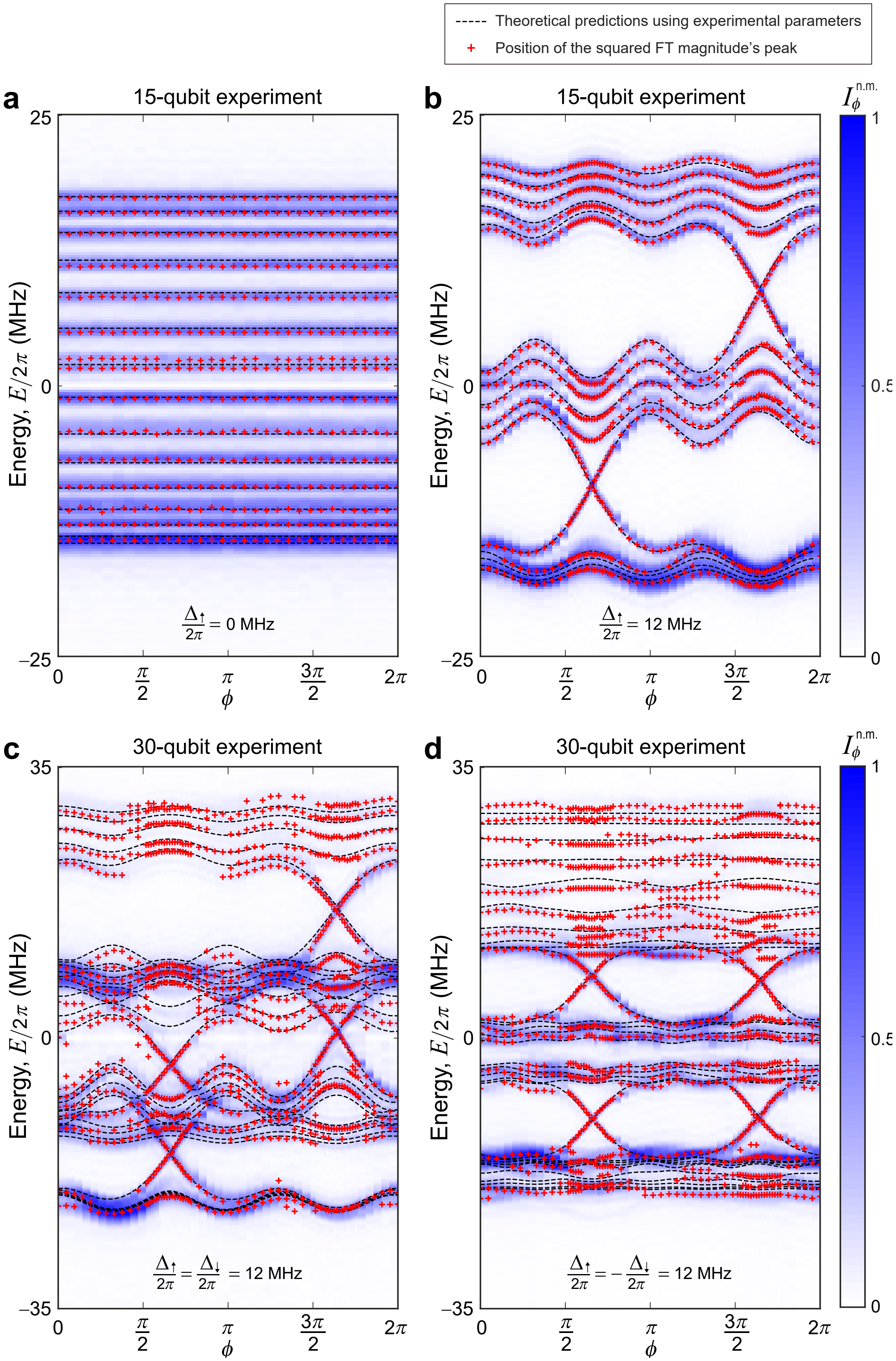

Supplementary Fig. 20. Comparison between the experimentally measured band structures by summing the FT magnitudes of all selected qubits (normalised data $I_\phi^{\text{n.m.}}$) with the theoretical predictions using experimental parameters. (a) is for the 15-qubit experiment with $\Delta_\uparrow/2\pi = 0$ MHz. (b) is for the 15-qubit experiment with $\Delta_\uparrow/2\pi = 12$ MHz and $b = \frac{1}{3}$. (c) is for the 30-qubit experiment with $\Delta_\uparrow/2\pi = \Delta_\downarrow/2\pi = 12$ MHz and $b = \frac{1}{3}$. (d) is for the 30-qubit experiment with $\Delta_\uparrow/2\pi = -\Delta_\downarrow/2\pi = 12$ MHz and $b = \frac{1}{3}$.

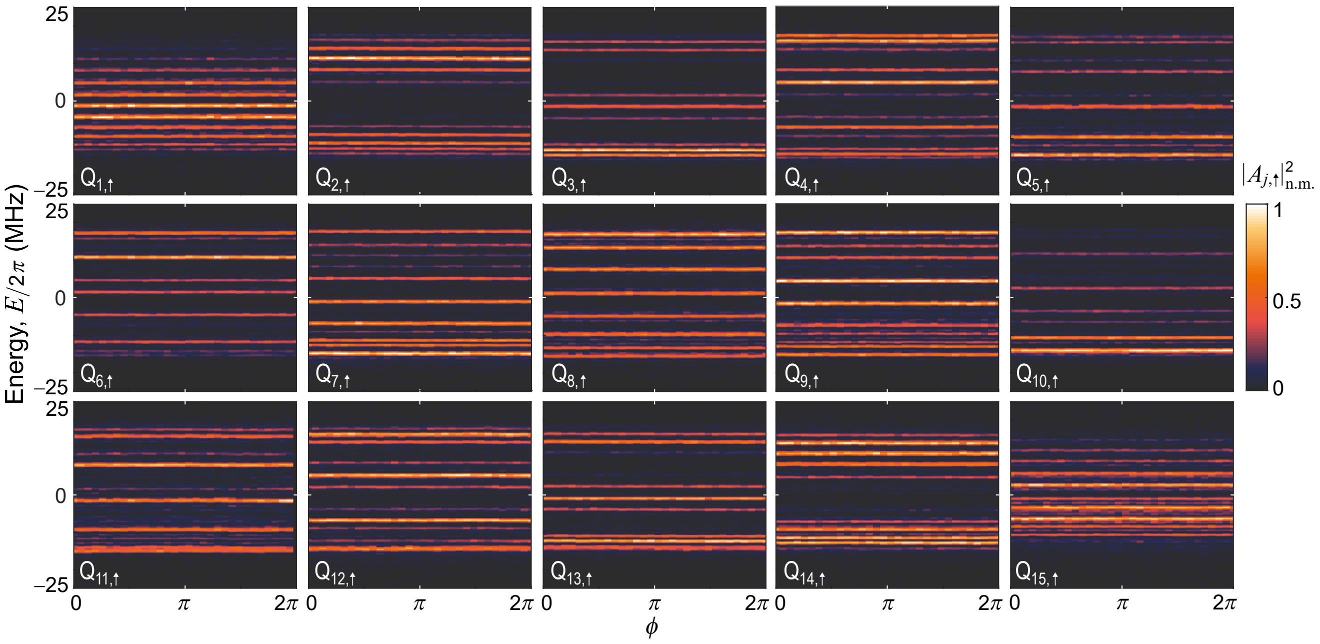

Supplementary Fig. 21. Experimental data of the normalised squared FT magnitudes $|A_{j,\uparrow}|^2_{\mathrm{n.m.}}$ when choosing $\mathrm{Q}_{j,\uparrow}$ as the target qubit in the 15-qubit experiment with $\Delta_{\uparrow}/2\pi = 0$ MHz.

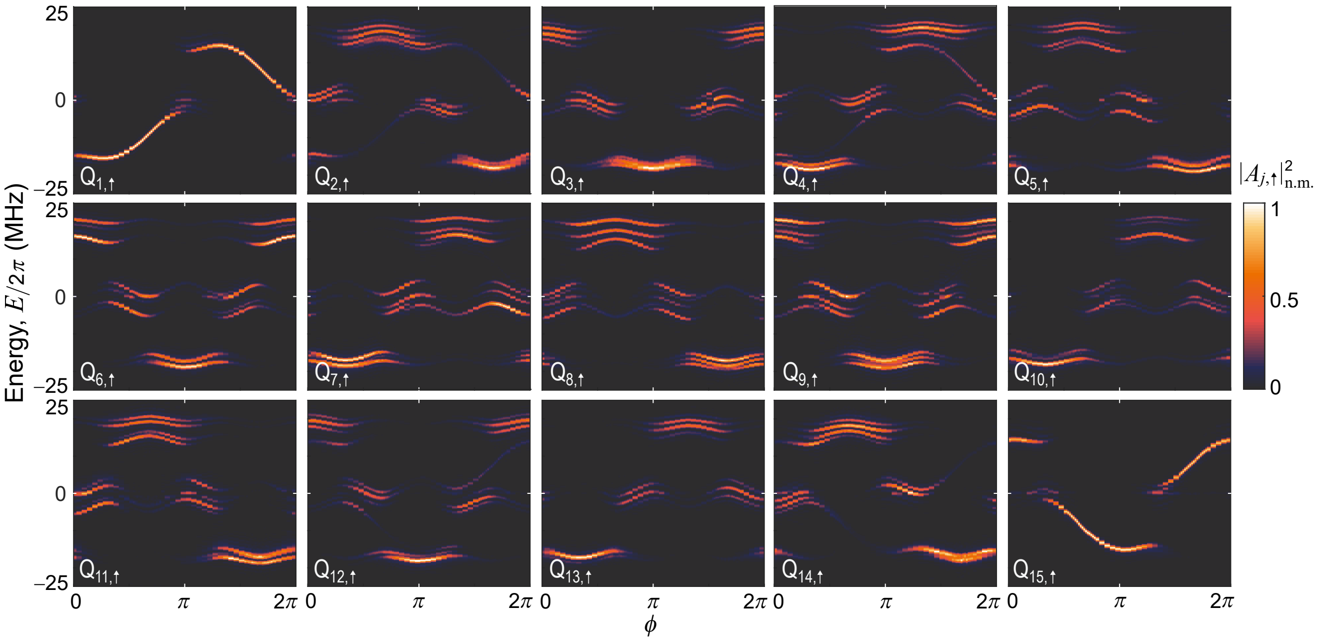

Supplementary Fig. 22. Experimental data of the normalised squared FT magnitudes $|A_{j,\uparrow}|^2_{\mathrm{n.m.}}$ when choosing $\mathrm{Q}_{j,\uparrow}$ as the target qubit in the 15-qubit experiment with $b = \frac{1}{3}$ and $\Delta_{\uparrow}/2\pi = 12$ MHz.

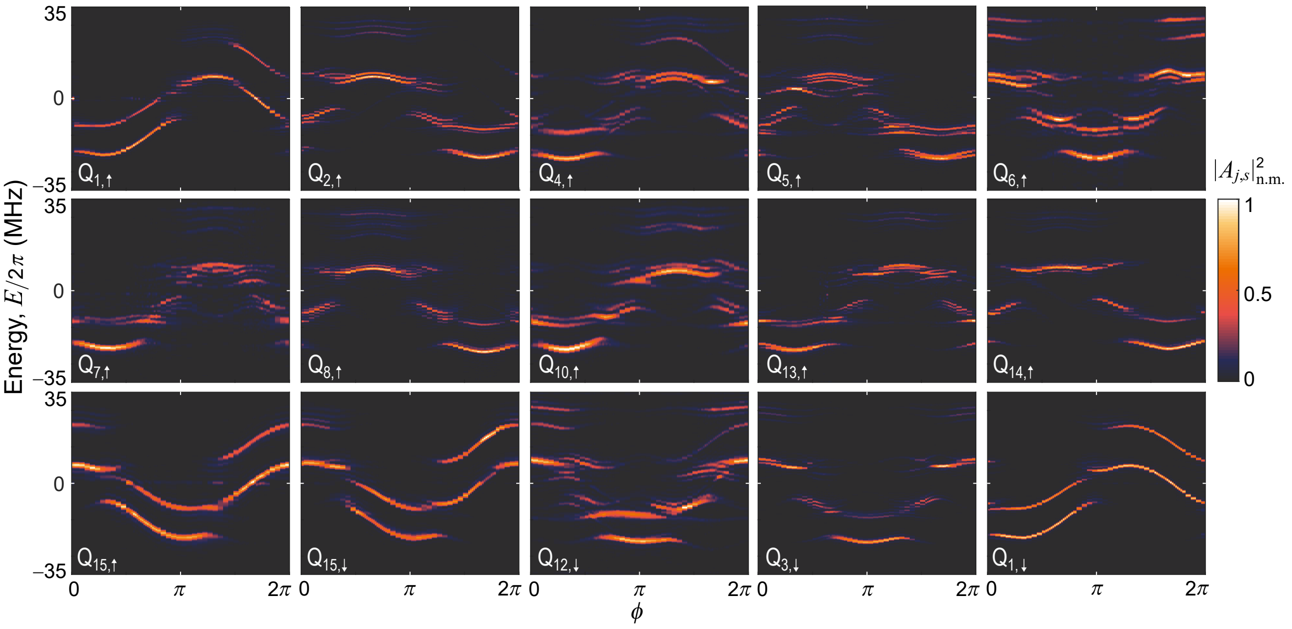

Supplementary Fig. 23. Experimental data of the normalised squared FT magnitudes $|A_{j,s}|^2_{\text{n.m.}}$ when choosing $Q_{j,s}$ as the target qubit in the 30-qubit experiment with $\Delta_\uparrow/2\pi = \Delta_\downarrow/2\pi = 12$ MHz and $b = \frac{1}{3}$.

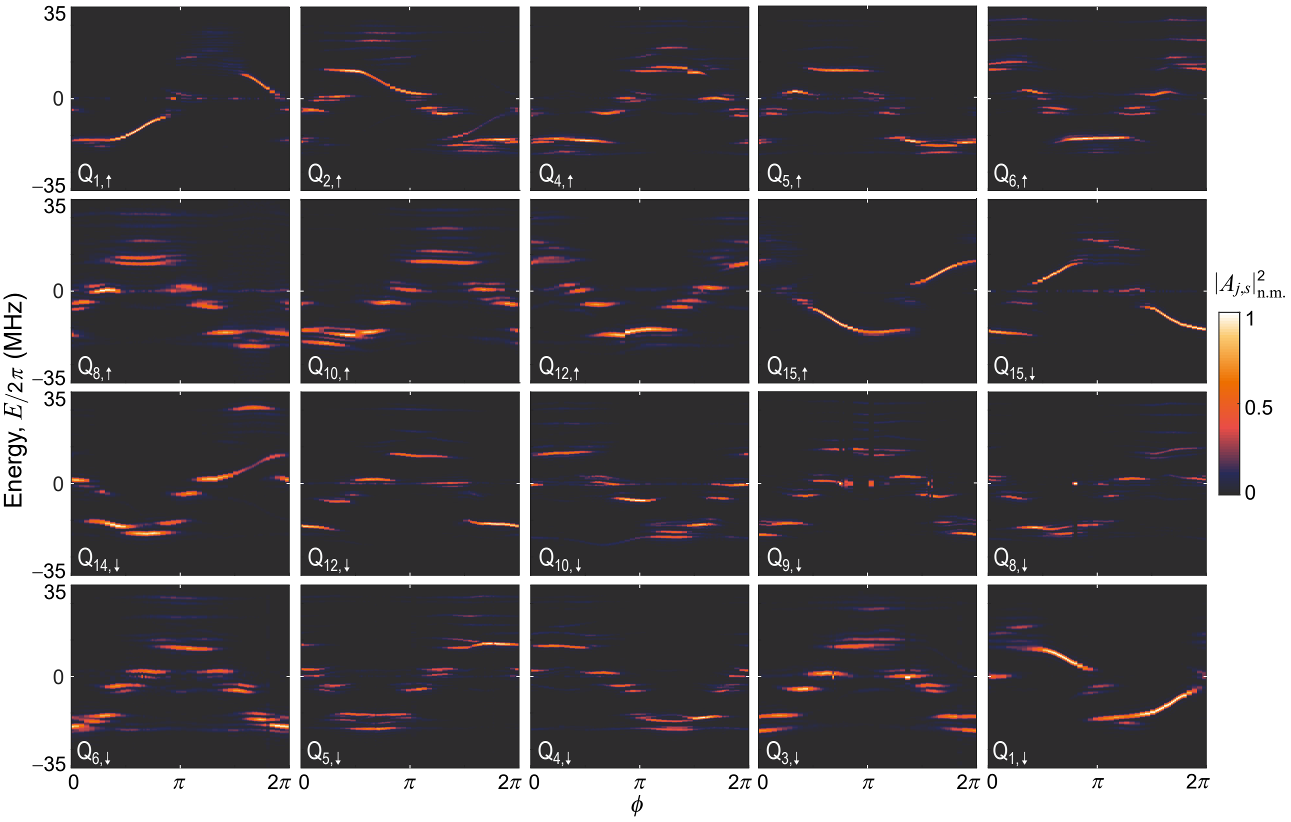

Supplementary Fig. 24. Experimental data of the normalised squared FT magnitudes $|A_{j,s}|^2_{\text{n.m.}}$ when choosing $Q_{j,s}$ as the target qubit in the 30-qubit experiment with $\Delta_\uparrow/2\pi = -\Delta_\downarrow/2\pi = 12$ MHz and $b = \frac{1}{3}$.

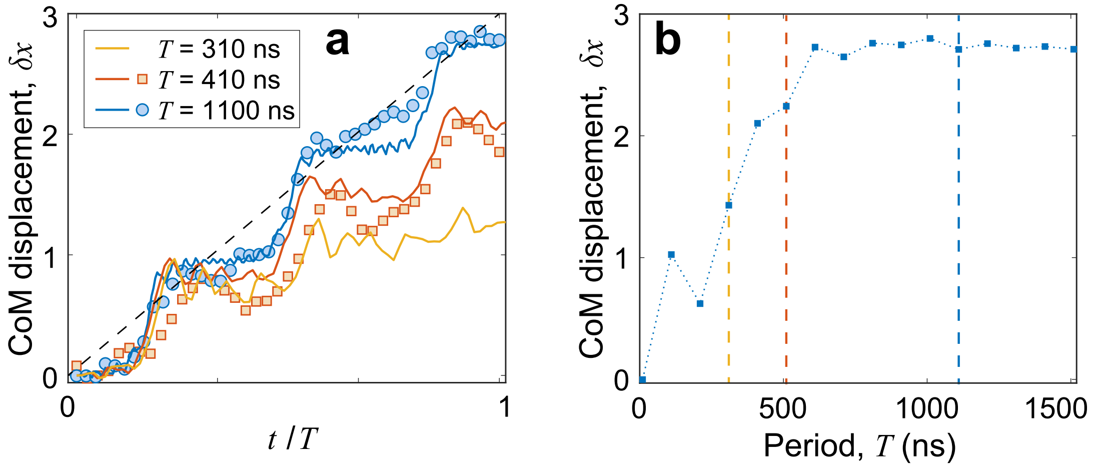

Supplementary Fig. 25. **a,** Experimental data (circles and squares for different periods $T = 410$ ns and 1,100 ns, respectively) for the displacement of the centre of mass (CoM) δx versus time t within one pumping cycle, compared with the numerical simulations (solid curves) with different periods $T = 310$ ns, 410 ns, and 1,100 ns. **b,** Numerical data for the displacement of the CoM δx in one pumping cycle versus different pumping periods T.

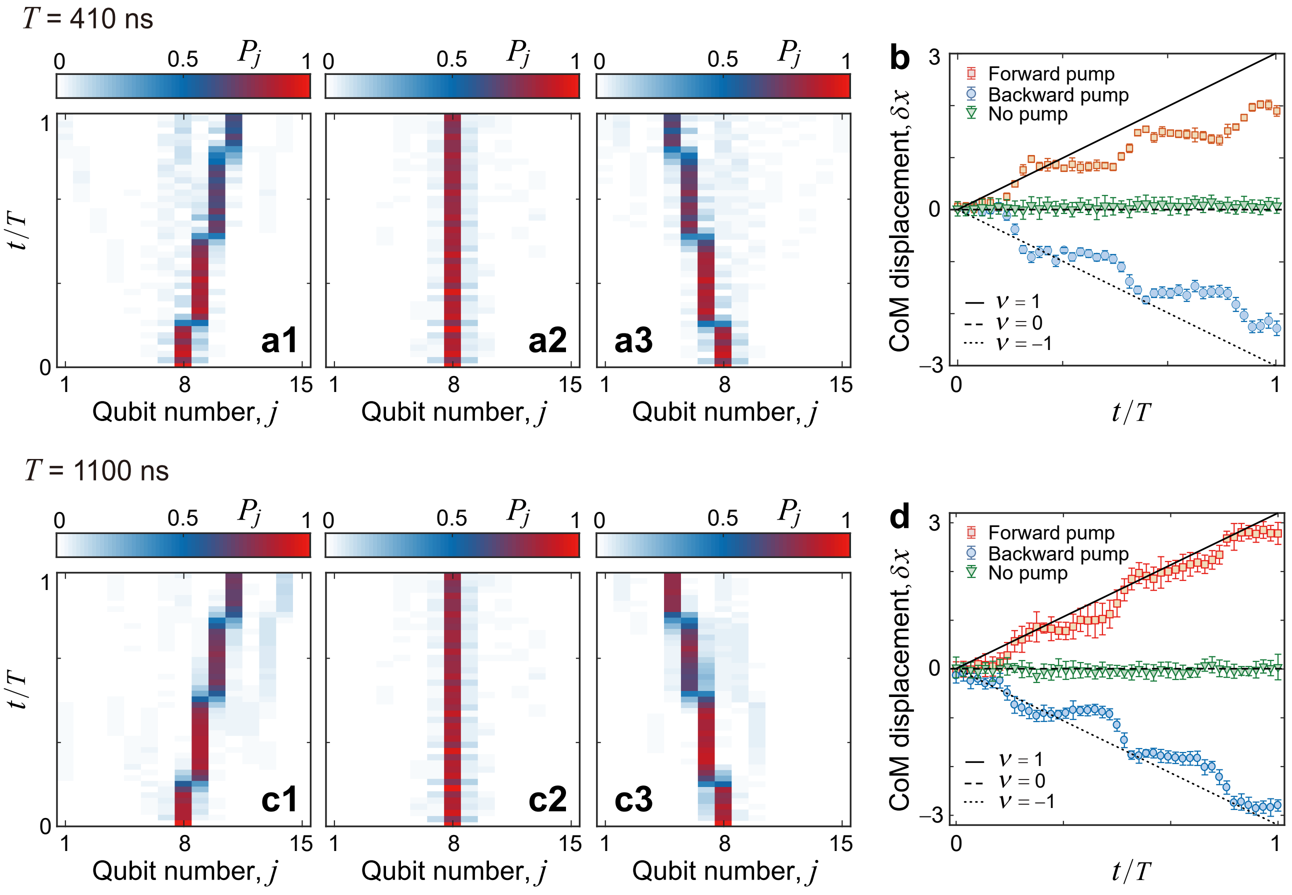

Supplementary Fig. 26. **a1–a3**, Time evolution of an excitation initially prepared at the central qubit Q_8 when it is forward pumped (**a1**), not pumped (**a2**) and backward pumped (**a3**), respectively, with $T = 410$ ns and $\Delta/2\pi = 36$ MHz for an initial $\phi_0 = 5\pi/3$. **b**, Displacement of the centre of mass (CoM) δx versus time t in one pumping cycle with period T for the cases in (**a1–a3**). The error bars are 1 SD, calculated from all groups of experimental results. **c1–c3**, Time evolution of an excitation initially prepared at the central qubit Q_8 when it is forward pumped (**c1**), not pumped (**c2**) and backward pumped (**c3**), respectively, with $T = 1,100$ ns and $\Delta/2\pi = 36$ MHz for an initial $\phi_0 = 5\pi/3$. **d**, Displacement of the CoM δx versus time t in one pumping cycle with period T for the cases in (**c1–c3**). The error bars are 1 SD, calculated from 10 groups of experimental measurement results.
