# Supplementary material for: Simulating Chern insulators on a superconducting quantum processor
Source: Nat Commun. 2023 Sep 5;14:5433. doi: 10.1038/s41467-023-41230-9 (PMC10480218; doi:10.1038/s41467-023-41230-9)
Supplement: Supplementary file 3 — Description of Additional Supplementary Files [file 41467_2023_41230_MOESM3_ESM.docx]

**Description of Additional Supplementary Files**

**File Name: Supplementary Movie 1
Description:** a-c, Animations for Fig. 4e-g, showing time evolutions of the excitation probability after exciting a corner qubit.
